# Comparison of Clinical, Metabolic, and Hormonal Profiles in Obese Versus Lean Women With Polycystic Ovary Syndrome: A Systematic Review and Meta‐Analysis

**DOI:** 10.1002/edm2.70350

**Published:** 2026-10-08

**Authors:** Fatema Tuz Zahura Aalpona, Apurba Sarker, Lutfun Nahar, Md Abdul Bari Robel, A. B. M. Kamrul‐Hasan

**Affiliations:** ^1^ Department of Gyne & Obs Mymensingh Medical College Mymensingh Bangladesh; ^2^ Department of Endocrinology Mymensingh Medical College Hospital Mymensingh Bangladesh; ^3^ Department of Endocrinology Cumilla Medical College Hospital Cumilla Bangladesh; ^4^ Department of Endocrinology Mymensingh Medical College Mymensingh Bangladesh

**Keywords:** body mass index, cardiometabolic risk, hyperandrogenism, insulin resistance, obesity, polycystic ovary syndrome

## Abstract

**Introduction:**

Obesity substantially influences clinical and metabolic manifestations of polycystic ovary syndrome (PCOS). However, differences in clinical, metabolic, and hormonal profiles between obese and lean women with PCOS have not been explored in large‐scale meta‐analyses.

**Methods:**

Databases were searched from inception through 31 December 2025 for studies reporting clinical, metabolic, and hormonal profiles in obese and lean PCOS phenotypes. Random‐effects meta‐analyses in R yielded mean differences (MDs) or risk ratios (RRs) with 95% confidence intervals.

**Results:**

Forty‐two studies (*N* = 5363; 3078 obese, 2285 lean PCOS) met inclusion criteria. Menstrual irregularity (84.7% vs. 70.9%; RR 1.19), hirsutism (65.9% vs. 58.8%; RR 1.10), acne (44.9% vs. 36.8%; RR 1.25), acanthosis nigricans (52.7% vs. 22.6%; RR 1.94), and biochemical hyperandrogenism (45.6% vs. 31.8%; RR 1.49) were more prevalent in obese than in lean PCOS. Obese PCOS was associated with a less favourable cardiometabolic profile, with higher HOMA‐IR (MD 2.03), fasting (MD 5.12 mg/dL), and 2‐h (MD 9.91 mg/dL) plasma glucose, and greater risks of prediabetes (25.3% vs. 12.1%; RR 1.84), diabetes (8.6% vs. 2.7%; RR 2.58), and metabolic syndrome (51.2% vs. 11.9%; RR 3.59). Mean lipid levels were worse in obese PCOS, with higher total cholesterol, triglycerides, and LDL cholesterol, and lower HDL cholesterol. Nonetheless, lean PCOS exhibited substantial burdens of menstrual dysfunction, hyperandrogenism, insulin resistance, dysglycemia, and dyslipidemia.

**Conclusion:**

Obesity in PCOS is associated with more severe reproductive, metabolic, and hormonal abnormalities, but lean PCOS still carries substantial reproductive and metabolic burdens. However, the observational design of the included studies and substantial heterogeneity across several outcomes warrant cautious interpretation.

**Trial Registration:**

The meta‐analysis was registered with PROSPERO under registration number CRD420251240521. The review protocol summary is available on the PROSPERO website

## Graphical abstract

This meta‐analysis of 42 studies, including 5363 women with PCOS, found that obesity was associated with more severe reproductive, androgenic, metabolic, and cardiometabolic abnormalities. Lean PCOS also carried substantial metabolic risk, supporting phenotype‐informed assessment and cardiometabolic screening regardless of BMI.

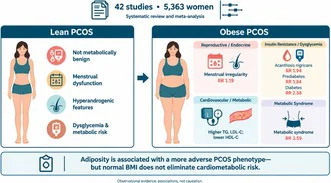

**Abbreviations**
2 h PG: 2‐h plasma glucose
BMI: body mass index
BP: blood pressure
CI: confidence interval
FPG: fasting plasma glucose
FSH: follicle‐stimulating hormone
HDL‐C: high‐density lipoprotein cholesterol
HOMA‐IR: homeostasis model assessment of insulin resistance
HTN: hypertension
IR: insulin resistance
JBI: Joanna Briggs Institute
LDL‐C: low‐density lipoprotein cholesterol
LH: luteinizing hormone
MD: mean difference
MeSH: Medical Subject Headings
mFG: modified Ferriman–Gallwey
NIH: National Institutes of Health
ORCID: Open Researcher and Contributor ID
PCOS: polycystic ovary syndrome
PICOS: Population, Intervention, Comparison, Outcomes, and Study design
PRISMA: Preferred Reporting Items for Systematic Reviews and Meta‐Analyses
PROSPERO: International Prospective Register of Systematic Reviews
RR: risk ratio
SRMA: systematic review and meta‐analysis
T2D: type 2 diabetes
TT: total testosterone
WHO: World Health Organization

## 1 Introduction

Polycystic ovary syndrome (PCOS) is one of the most prevalent endocrine disorders, affecting roughly 6%–20% of women of reproductive age, with a highly varied presentation [1]. Diagnosis is usually made based on the revised Rotterdam criteria, which require at least two of the following three features: clinical or biochemical hyperandrogenism, ovulatory dysfunction, and polycystic ovarian morphology on ultrasound [2]. Overweight or obesity frequently accompanies PCOS, exacerbating the clinical and metabolic manifestations of the syndrome, although many patients with PCOS are lean [3]. This phenotypic diversity has prompted comparative studies of lean and obese PCOS phenotypes to explore distinct pathophysiological mechanisms. It is well‐established that women with PCOS, especially those who have excess weight, more frequently experience IR and compensatory hyperinsulinemia, which in turn promotes increased androgen production and other metabolic dysregulation, including dyslipidemia and a higher risk of developing type 2 diabetes (T2D) [4, 5]. Lean women with PCOS may have a different background for their pathogenesis, driven more by inherent hormonal dysregulation, such as high frequency or amplitude of luteinizing hormone (LH) pulses, or functional ovarian hyperandrogenism, than by adiposity‐driven metabolic insults [6].

However, the literature is inconsistent, yielding conflicting findings. Several reports suggest that lean women with PCOS have a milder metabolic profile but a similarly disturbed reproductive hormonal profile, while some indicate that obesity is a major factor driving all PCOS manifestations and that lean patients constitute a distinct, less severe subgroup [7, 8]. Therefore, this inconsistency presents a dilemma for the clinician in prognostication and management according to phenotype. Although several narrative reviews have covered the topic, a full quantitative summary of the evidence is lacking. A systematic review and meta‐analysis (SR/MA) comparing clinical features such as hirsutism, acne, and acanthosis nigricans, and metabolic parameters including glucose, lipid profile, homeostasis model assessment for insulin resistance (HOMA‐IR), and hormonal profiles between lean and obese women with PCOS is needed to clarify these phenotypic differences. This SR/MA aimed to quantitatively compare these phenotypic differences and inform phenotype‐specific clinical assessment.

## 2 Methods

### 2.1 Study Registration and Reporting

This SR/MA followed procedures from the Cochrane Handbook for Systematic Reviews of Interventions and is reported in accordance with the Preferred Reporting Items for Systematic Reviews and Meta‐Analyses (PRISMA) checklist [9, 10]. It is registered with PROSPERO (CRD420251240521), and the protocol summary is available online.

### 2.2 Search Strategy

A comprehensive search was conducted across multiple databases and registries, including PubMed, Scopus, and Web of Science. Google Scholar was also utilized to find additional articles. The search included all sources from their inception through 31 December 2025. Using the Boolean operators “AND” and “OR,” the following terms were searched: “polycystic ovary syndrome,” “polycystic ovarian syndrome,” “PCOS,” “obesity,” “obese,” “overweight,” “body mass index,” “BMI,” “lean,” “normal weight,” “non‐obese,” “nonobese,” “clinical characteristics,” “clinical,” “symptom,” “sign,” “metabolic diseases,” “metabolic,” “insulin resistance,” “glucose,” “lipid,” “dyslipidemia,” “hormones,” “hormonal,” “androgen,” “testosterone,” “luteinizing hormone,” “LH,” “follicle stimulating hormone,” and “FSH.” The search used MeSH terms, document titles, abstracts, and keywords, with no language restrictions, to identify human studies. Appendix S1 provides the full set of search strategies. The process also included reviewing references cited in the collected articles and in relevant journals.

The review was registered and conducted using the term “polycystic ovary syndrome” (PCOS), as specified in the prespecified protocol, search strategy, diagnostic frameworks, and all included reports. Although polyendocrine metabolic ovarian syndrome (PMOS) has since been proposed as the preferred name for the condition formerly known as PCOS, we retained “PCOS” in this review to maintain consistency with the source literature and facilitate reproducibility of the search and study‐selection process.

### 2.3 Study Selection

The eligibility criteria were structured using the Population, Exposure, Comparator, Outcomes, and Study design (PECOS) framework. The population (P) comprised women with PCOS. The exposure group (E) comprised women with PCOS who had overweight or obesity, and the comparator group (C) comprised women with PCOS who were lean or had normal body weight. The measured outcomes (O) included clinical, metabolic, and hormonal profiles. The study types (S) included observational studies (cross‐sectional, case‐control, or cohort) and baseline data from randomized or nonrandomized clinical trials. Animal studies, case reports, case series, review articles, or articles that did not report the specified outcomes were excluded. Three review authors independently selected eligible articles according to the criteria outlined above; details are provided elsewhere [11].

### 2.4 Outcomes Analysed

We considered all reported clinical, metabolic, and hormonal profiles as outcomes for this SR/MA. The outcomes included menstrual irregularity, the modified Ferriman–Gallwey (mFG) score, hirsutism, acne, family history of T2D, acanthosis nigricans, systolic and diastolic blood pressure (BP), hypertensive status, fasting plasma glucose (FPG), 2‐h plasma glucose (2 h PG) (defined as plasma glucose measured 2 h after either an oral glucose tolerance test or breakfast and pooled as a post‐challenge/postprandial glycemic outcome), glycemic status, HOMA‐IR, fasting lipid profile, metabolic syndrome, total testosterone (TT), and biochemical hyperandrogenism (defined according to each study's pre‐specified thresholds for elevated TT).

### 2.5 Data Extraction and Handling Missing Data

Data from all eligible studies included the first author, publication year, study type, country, key inclusion criteria, BMI cutoff for overweight/obesity, and the number and mean BMI of participants in both groups. Additionally, data on the specified outcomes were collected. Details of the data extraction procedure and management of missing data are available in a separate source [11]. Potentially overlapping populations were assessed by comparing author names, institutions, recruitment periods, sample sizes, participant characteristics, and study descriptions. When multiple reports appeared to originate from the same study population, they were treated as a single study, and outcome data were extracted only once per meta‐analysis to avoid double counting.

### 2.6 Assessment of the Quality of the Included Studies

Two authors independently evaluated the methodological quality of the included studies using the Joanna Briggs Institute (JBI) critical appraisal checklist for analytical cross‐sectional studies [12]. This checklist comprises eight key questions designed to assess the validity and reliability of each study. Reviewers answer each item with “Yes,” “No,” “Unclear,” or “Not Applicable.” These responses inform an overall decision to “Include,” “Exclude,” or “Seek Further Info” for the systematic review [12]. Discrepancies were resolved through consensus.

### 2.7 Statistical Analysis

Statistical analyses were conducted using the RStudio software environment (Version 2026.01.1+403, released on 19 February 2026), specifically utilizing the “meta” and “metafor” packages, which are designed for performing meta‐analyses in R [13]. The effect estimates were reported as MDs for continuous variables and risk ratios (RRs) for dichotomous variables, with 95% confidence intervals (CIs). Forest plots were generated using the same software to present MD or RR estimates, with effects plotted to the left of the line of no effect (MD = 0, RR = 1) indicating higher values in the lean PCOS group, and effects to the right indicating higher values in the obese PCOS group. The random‐effects model was selected to account for expected heterogeneity arising from population characteristics across the included studies. The inverse‐variance statistical method was used in all cases. The details of the meta‐analysis included the use of the restricted maximum‐likelihood estimator for *τ* ^2^, the Q‐profile method for the confidence interval of *τ* ^2^ and *τ*, and the calculation of *I* ^2^ based on Q statistics using untransformed (raw) means. A significance level of *p* < 0.05 was used.

### 2.8 Assessment of Heterogeneity and Methods Used to Explore Possible Causes of Heterogeneity Among Study Results

The heterogeneity assessment began with the analysis of forest plots. Next, Chi‐square tests were performed with N‐1 degrees of freedom at the 0.05 significance level, and the *I* ^2^ statistic was also used. *I* ^2^ thresholds were set at 25%, 50%, and 75% to indicate low, moderate, and high heterogeneity, respectively [14]. Possible sources of heterogeneity were explored using prespecified subgroup analyses. For outcomes with sufficient data, pooled estimates were stratified by BMI cutoff used to define obese PCOS (23–24.9 vs. ≥ 25 kg/m^2^), geographic region/continent (Asian vs. non‐Asian studies), and PCOS diagnostic criteria (Rotterdam or modified Rotterdam vs. non‐Rotterdam/NIH/local criteria). Between‐subgroup differences were assessed statistically. No formal meta‐regression was performed; therefore, residual heterogeneity related to continuous study‐level characteristics (e.g., age, baseline BMI, or assay methods) was not evaluated.

### 2.9 Publication Bias

To evaluate publication bias, funnel plots and Egger's regression test were conducted in RStudio when at least 10 studies were included in the forest plots [13, 15, 16].

## 3 Results

### 3.1 Search Results

The initial search identified 5725 records from databases/registers and 7280 from other sources; of the database/registry records, 1145 duplicates were removed, and 4580 titles and abstracts were screened, of which 4541 records were excluded. 73 reports (39 from databases/registers and 34 from other sources) were sought (with 3 not retrieved); 70 full texts were assessed for eligibility across the two streams, and 27 reports were excluded for predefined reasons (e.g., wrong population, inappropriate design, insufficient data). Finally, 42 studies (43 reports) with 5363 subjects (obese group: 3078; lean group: 2285) were included in the SR/MA, as shown in the PRISMA 2020 flow diagram (Figure 1) [17, 18, 19, 20, 21, 22, 23, 24, 25, 26, 27, 28, 29, 30, 31, 32, 33, 34, 35, 36, 37, 38, 39, 40, 41, 42, 43, 44, 45, 46, 47, 48, 49, 50, 51, 52, 53, 54, 55, 56, 57, 58, 59]. The two reports by Akshaya and Bhattacharya were considered complementary reports from the same study population and were grouped as one study; outcome data were extracted only once for each meta‐analysis [17, 18].

**FIGURE 1:**
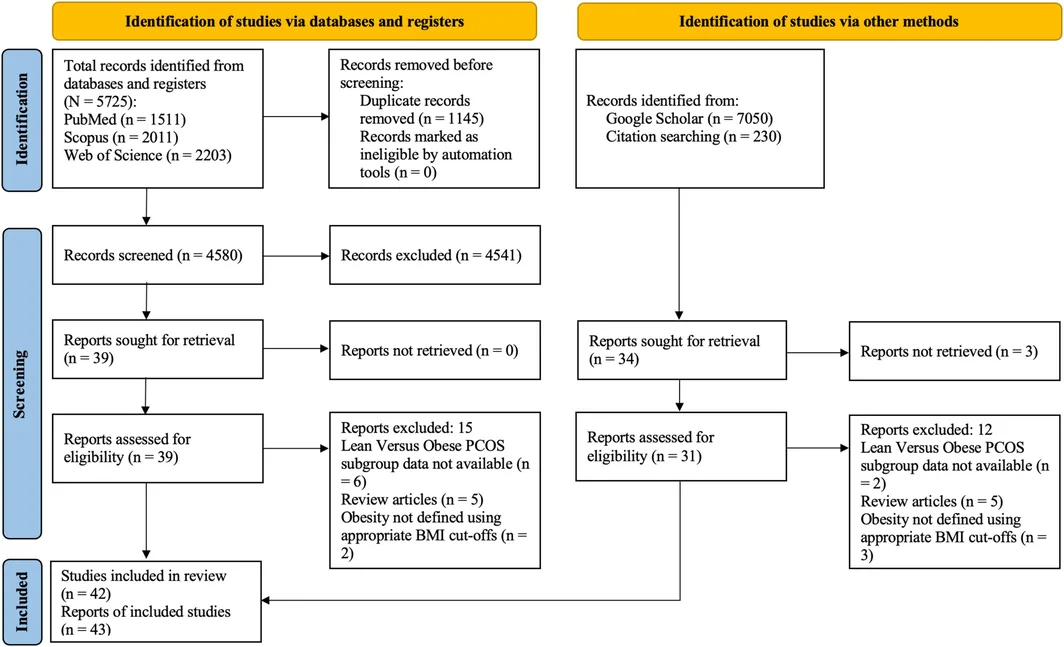
PRISMA 2020 flow diagram for new systematic reviews, which included searches of databases, registers, and other sources.

### 3.2 Characteristics of Included Studies

The specifics of the included studies are shown in Table S1. Most included studies were observational cross‐sectional or comparative cross‐sectional designs conducted in endocrine or gynaecology clinics in India [17, 18, 19, 20, 25, 27, 28, 29, 32, 34, 37, 43, 44, 45, 46, 49, 51, 52], China or Taiwan [39, 40, 55], Bangladesh [33], Iran [26, 38, 54], Iraq [30, 31, 47], and Saudi Arabia [42, 50, 56, 58], with additional data from Croatia [21], Romania [23], Greece [53], Poland [36], South Africa [41], the United Kingdom [35], United States [22, 57], Kyrgyzstan [48], and the Philippines [59]. Across studies, participants were predominantly reproductive‐aged women with PCOS diagnosed using Rotterdam or modified Rotterdam criteria [17, 18, 19, 20, 21, 22, 25, 26, 27, 28, 29, 30, 31, 32, 33, 34, 36, 37, 38, 39, 40, 43, 44, 46, 47, 48, 49, 50, 51, 52, 54, 55, 56, 59], with a minority using NIH‐ or Endocrine Society‐based criteria or local ultrasound‐driven definitions [24, 35, 41, 42, 45, 53, 58]. For adolescent‐only or adolescent‐focused PCOS cohorts, dedicated studies from Romania, India, Bangladesh, and the United States focused on girls aged approximately 11–20 years, applying adolescent‐specific adaptations of PCOS diagnostic criteria [20, 23, 33, 57]. Sample sizes ranged from small single‐center cohorts (*n* ≈ 40–60) to large series exceeding 200 women. Across the included studies, participants were stratified into lean (or non‐obese) and obese subgroups using varying BMI thresholds ranging from 23 to 30 kg/m^2^, reflecting both Asian‐specific and WHO obesity criteria. The most commonly applied thresholds were 23 kg/m^2^ [17, 18, 20, 28, 29, 32, 33, 34, 43, 48, 49, 52], 25 kg/m^2^ [21, 24, 26, 27, 30, 35, 36, 37, 38, 39, 40, 42, 44, 50, 51, 54, 56, 57], and 30 kg/m^2^ [31, 45, 47, 58]. Additional intermediate cut‐offs included 23.5 kg/m^2^ [25], 24 kg/m^2^ [41], 27 kg/m^2^ [46], and 28 kg/m^2^ [55], reflecting regional adaptations of BMI classification criteria. Mean age generally lay in the early to mid‐twenties, with baseline BMI spanning the normal‐to‐obese range across lean and obese strata. The mean age (MD 0.89 years, 95% CI [0.43, 1.35]), BMI (MD 8.52 kg/m^2^, 95% CI [7.79, 9.26]), waist circumference (MD 19.84 cm, 95% CI [16.51, 23.16]), and waist‐to‐hip ratio (MD 0.08, 95% CI [0.05, 0.11]) were higher in the obese group than in the lean group (Figures S1–S4, respectively).

### 3.3 Quality and Risk of Bias of the Included Studies

Across the 42 analytical cross‐sectional studies, most met the JBI criteria for clearly defined inclusion criteria, valid exposure measurement, use of standard diagnostic criteria for PCOS, valid outcome measurement, and appropriate basic statistical analysis (predominantly “Yes” ratings on items 1, 3, 4, 7, and 8) (Table S2). In contrast, almost none of the studies explicitly identified potential confounding factors or reported strategies to address them, and many provided only limited detail on the study setting and recruitment procedures, indicating a consistent methodological weakness around confounding control and, to a lesser extent, external validity. Overall, the evidence has moderate methodological quality, with strengths in case definition and measurement but systematic shortcomings in accounting for confounding.

### 3.4 Menstrual Irregularity

The proportion of subjects having menstrual irregularity (20 studies, *n* = 3641) was higher in the obese PCOS (84.7%) than the lean PCOS (70.9%) group (RR 1.19 [1.08; 1.32], *p* = 0.0008, *I* ^2^ = 86.2%) (Table 1).

**TABLE 1:** Comparison of clinical, metabolic, and hormonal profiles in lean vs. obese women with polycystic ovary syndrome.

Outcome variables	No. of study reports	*N*	Proportion of subjects with outcome (no. of with outcome/no. of participants)		Pooled effect size, RR (95% CI)	*p*	*I* ^2^ (%)
			Obese PCOS	Lean PCOS			
Menstrual irregularity	20	3641	84.7% (1755/2072)	70.9% (1112/1569)	1.19 [1.08; 1.32]	0.0008	86.2
Hirsutism	23	3659	65.9% (1367/2073)	58.8% (933/1586)	1.10 [1.01; 1.20]	0.0323	63.8
Acne	19	2433	44.9% (610/1358)	36.8% (396/1075)	1.25 [1.03; 1.51]	0.0215	62.4
Family history of T2D	6	863	29.7% (179/603)	22.3% (58/260)	1.47 [1.04; 2.08]	0.0292	26.8
Acanthosis nigricans	15	1689	52.7% (570/1082)	22.6% (137/607)	1.94 [1.68; 2.25]	< 0.0001	0
Biochemical hyperandrogenism	7	1883	45.6% (514/1126)	31.8% (241/757)	1.49 [1.01; 2.20]	0.0435	80.9
HTN	6	1432	14.2% (147/1036)	5.8% (23/396)	2.27 [1.11; 4.66]	0.0255	52.4
Insulin resistance	5	479	68.3% (205/300)	55.3% (99/179)	1.16 [1.02; 1.32]	0.0261	4.9
Prediabetes	10	1747	25.3% (298/1176)	12.1% (69/571)	1.84 [1.41; 2.39]	< 0.0001	0
Diabetes	9	1692	8.6% (101/1168)	2.7% (14/524)	2.58 [1.52; 4.38]	0.0004	0
Dyslipidemia	4	853	78.7% (477/606)	60.3% (149/247)	1.33 [0.95; 1.87]	0.0987	73.5
Metabolic syndrome	4	947	51.2% (360/703)	11.9% (29/244)	3.59 [2.50; 5.17]	< 0.0001	33.2

Abbreviations: CI, confidence interval; HTN, hypertension; PCOS, polycystic ovary syndrome; RR, risk ratio; T2D, type 2 diabetes.

### 3.5 Modified Ferriman–Gallwey Score and Hirsutism

mFG score (9 studies, *n* = 1152) was similar in the obese PCOS and in the lean PCOS group (MD 1.18 [−0.76; 3.13], *p* = 0.2337, *I* ^2^ = 78.6%) (Figure 2A). The MDs in the mFG score were similar between the obese and lean groups across study subgroups, according to the BMI cutoffs (23–24.9 versus 25 kg/m^2^) used to define obese PCOS (*P* for subgroup difference = 0.6486) (Figure S5), and across Asian versus Non‐Asian studies (*P* for subgroup difference = 0.6302) (Figure S6). The proportion of subjects with hirsutism (23 studies, *n* = 3659) was higher in the obese PCOS group (65.9%) than in the lean PCOS group (58.8%) (RR 1.10 [1.01; 1.20], *p* = 0.0323, *I* ^2^ = 63.8%) (Table 1).

**FIGURE 2:**
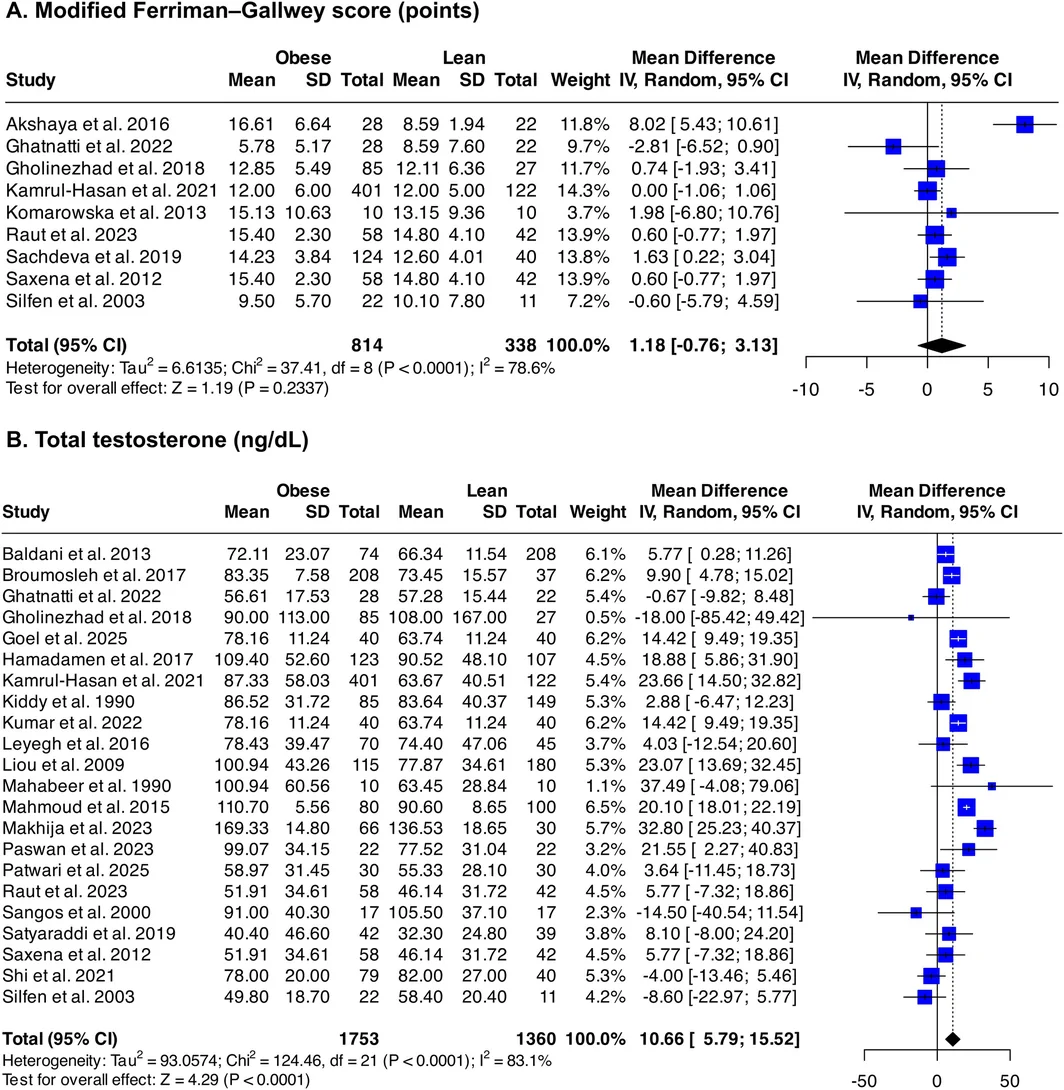
Forest plots of clinical and biochemical hyperandrogenism‐related outcomes comparing obese versus lean women with polycystic ovary syndrome (PCOS). (A) Modified Ferriman–Gallwey (mFG) score, expressed as points. (B) Total testosterone, expressed as ng/dL. Pooled mean differences (MDs) were calculated using random‐effects inverse‐variance meta‐analysis and are presented with 95% confidence intervals (CIs). The vertical line of no effect represents MD = 0. Estimates to the right of the line indicate higher values in the obese‐PCOS group, whereas estimates to the left indicate higher values in the lean‐PCOS group.

### 3.6 Acne

More subjects in the obese PCOS (44.9%) group had acne (19 studies, *n* = 2433) than in the lean PCOS (36.8%) group (RR 1.25 [1.03; 1.51], *p* = 0.0215, *I* ^2^ = 62.4%) (Table 1).

### 3.7 Family History of T2D

Family history of T2D (6 studies, *n* = 863) was more frequent in the obese PCOS group (29.7%) than in the lean PCOS group (22.3%) (RR 1.47 [1.04; 2.08], *p* = 0.0292, *I* ^2^ = 26.8%) (Table 1).

### 3.8 Acanthosis Nigricans

More subjects in the obese PCOS (52.7%) group had acanthosis nigricans (15 studies, *n* = 1689) than in the lean PCOS (22.6%) group (RR 1.94 [1.68; 2.25], *p* < 0.0001, *I* ^2^ = 0%) (Table 1).

### 3.9 Total Testosterone and Biochemical Hyperandrogenism

The obese PCOS group had higher TT (22 studies, *n* = 3113) than the lean PCOS group (MD 10.66 ng/dL [5.79; 15.52], *p* < 0.0001, *I* ^2^ = 83.1%) (Figure 2B). In the subgroup analysis, TT was significantly higher in the obese PCOS group only in studies using a BMI cutoff of 25 kg/m^2^ to define obese PCOS (*p* < 0.0001). In studies using BMI cutoffs of 23–24.9 and > 25 kg/m^2^, or those that did not report a cutoff, no significant differences in TT levels were observed between obese and lean groups. However, the between‐subgroup difference in MDs of TT was not statistically significant (*p* = 0.6230) (Figure S7). In continent‐based subgroups, TT was significantly higher in obese than in lean women with PCOS in Asian studies, but not in non‐Asian studies, and the between‐continent difference was statistically significant (*p* = 0.0345) (Figure S8). In analyses stratified by diagnostic criteria, TT was significantly higher in obese than in lean PCOS in studies using Rotterdam or modified Rotterdam criteria, but not in those using non‐Rotterdam (NIH/local) criteria; however, the between‐criteria subgroup difference was not statistically significant (*p* = 0.6526) (Figure S9). The proportion of subjects with raised TT (7 studies, *n* = 1883) was also higher in the obese than in the lean PCOS (45.6% vs. 31.8%) group (RR 1.49 [1.01; 2.20], *p* = 0.0435, *I* ^2^ = 80.9%) (Table 1).

### 3.10 Blood Pressure and Hypertension

Both systolic BP (13 studies, *n* = 1700) and diastolic BP (13 studies, *n* = 1700) were higher in the obese group compared to the lean PCOS group (for systolic BP: MD 8.19 mmHg [5.74; 10.64], *p* < 0.0001, *I* ^2^ = 74.8% (Figure 3A); for diastolic BP: MD 6.17 mmHg [4.63; 7.72], *p* < 0.0001, *I* ^2^ = 66.4%; Figure 3B). More subjects in the obese than in the lean PCOS group (14.2% vs. 5.8%) had HTN (6 studies, *n* = 1432) (RR 2.27 [1.11; 4.66], *p* = 0.0255, *I* ^2^ = 52.4%) (Table 1).

**FIGURE 3:**
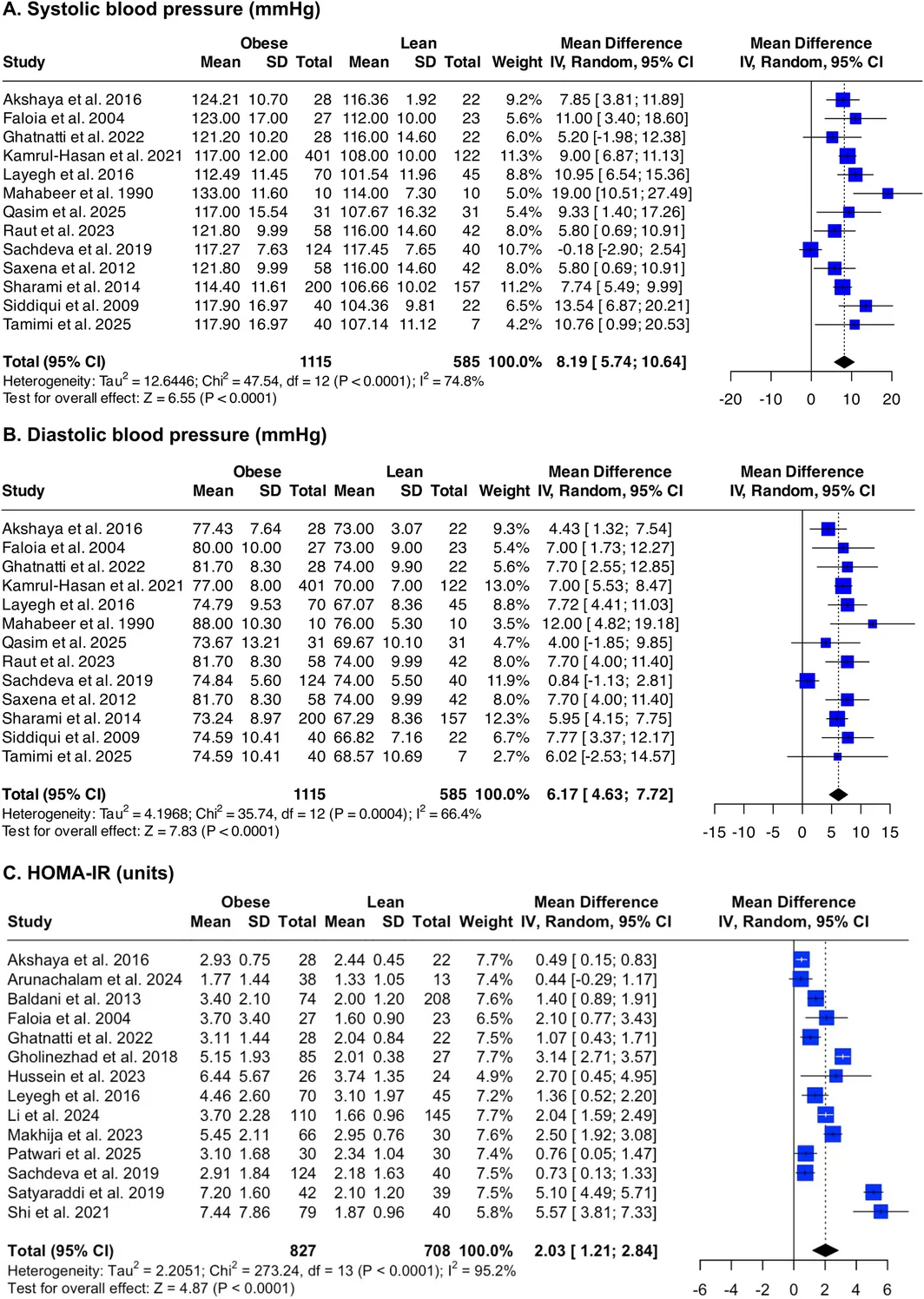
Forest plots of continuous cardiometabolic outcomes comparing obese versus lean women with polycystic ovary syndrome (PCOS). (A) Systolic blood pressure, expressed as mmHg. (B) Diastolic blood pressure, expressed as mmHg. (C) Homeostasis model assessment of insulin resistance (HOMA‐IR), expressed as units. Pooled mean differences (MDs) were calculated using random‐effects inverse‐variance meta‐analysis and are presented with 95% confidence intervals (CIs). The vertical line of no effect represents MD = 0. Estimates to the right of the line indicate higher values in the obese‐PCOS group, whereas estimates to the left indicate higher values in the lean‐PCOS group.

### 3.11 Homeostasis Model Assessment for Insulin Resistance

HOMA‐IR (14 studies, *n* = 1535) was higher in the obese group compared to the lean PCOS group (MD 2.03 [1.21; 2.84], *p* < 0.0001, *I* ^2^ = 95.2%; Figure 3C). In subgroup analyses by BMI cutoff, HOMA‐IR was significantly higher in obese than in lean women with PCOS across all BMI categories, with the largest mean difference observed in studies using a BMI cutoff of > 25 kg/m^2^; between‐subgroup differences were statistically significant (*p* = 0.0013) (Figure S10). In analyses stratified by continent, obese women with PCOS had higher HOMA‐IR than lean women in both Asian and non‐Asian studies, and the magnitude of this difference did not differ significantly between continents (*p* = 0.2706 for subgroup differences) (Figure S11). When stratified by PCOS diagnostic criteria, obese women consistently showed higher HOMA‐IR than lean women in both Rotterdam/modified Rotterdam and non‐Rotterdam (NIH/local) studies, with no significant difference between diagnostic subgroups (*p* = 0.7234 for subgroup differences) (Figure S12). More subjects in the obese than in the lean PCOS group (68.3% vs. 55.3%) had insulin resistance (5 studies, *n* = 479) (RR 1.16 [1.02; 1.32], *p* = 0.0261, *I* ^2^ = 4.9%) (Table 1).

### 3.12 Plasma Glucose and Glycemic Status

FPG (23 studies, *n* = 2659) and 2 h PG (18 studies, *n* = 2210) were higher in the obese than in the lean PCOS group (for FPG: MD 5.12 mg/dL [3.69; 6.55], *p* < 0.0001, *I* ^2^ = 58.2% (Figure 4A); for 2 h PG: MD 9.91 mg/dL [5.03; 14.79], *p* < 0.0001, *I* ^2^ = 84.7%; Figure 4B). The pooled 2 h PG outcome included measurements obtained after either an oral glucose tolerance test or breakfast, which may have contributed to heterogeneity. In subgroup analyses, FPG was consistently higher in obese than lean women with PCOS across all BMI cutoff and diagnostic‐criteria strata, and the magnitude of this difference did not differ significantly between BMI‐defined or diagnostic subgroups (*p* > 0.05 for both subgroup comparisons) (Figures S13 and S14, respectively). More subjects in the obese than in the lean PCOS group had prediabetes (10 studies, *n* = 1747) (25.3% vs. 12.1%; RR 1.84 [1.41; 2.39], *p* < 0.0001, *I* ^2^ = 0%), and diabetes (9 studies, *n* = 1692) (8.6% vs. 2.7%; RR 2.58 [1.52; 4.38], *p* = 0.0004, *I* ^2^ = 0%) (Table 1). In continent‐based subgroup analyses, the obese PCOS group had a higher prevalence of diabetes mellitus than the lean group in both Asian and non‐Asian studies, with similar effect sizes across continents (*p* = 0.3594 for subgroup differences) (Figure S15).

**FIGURE 4:**
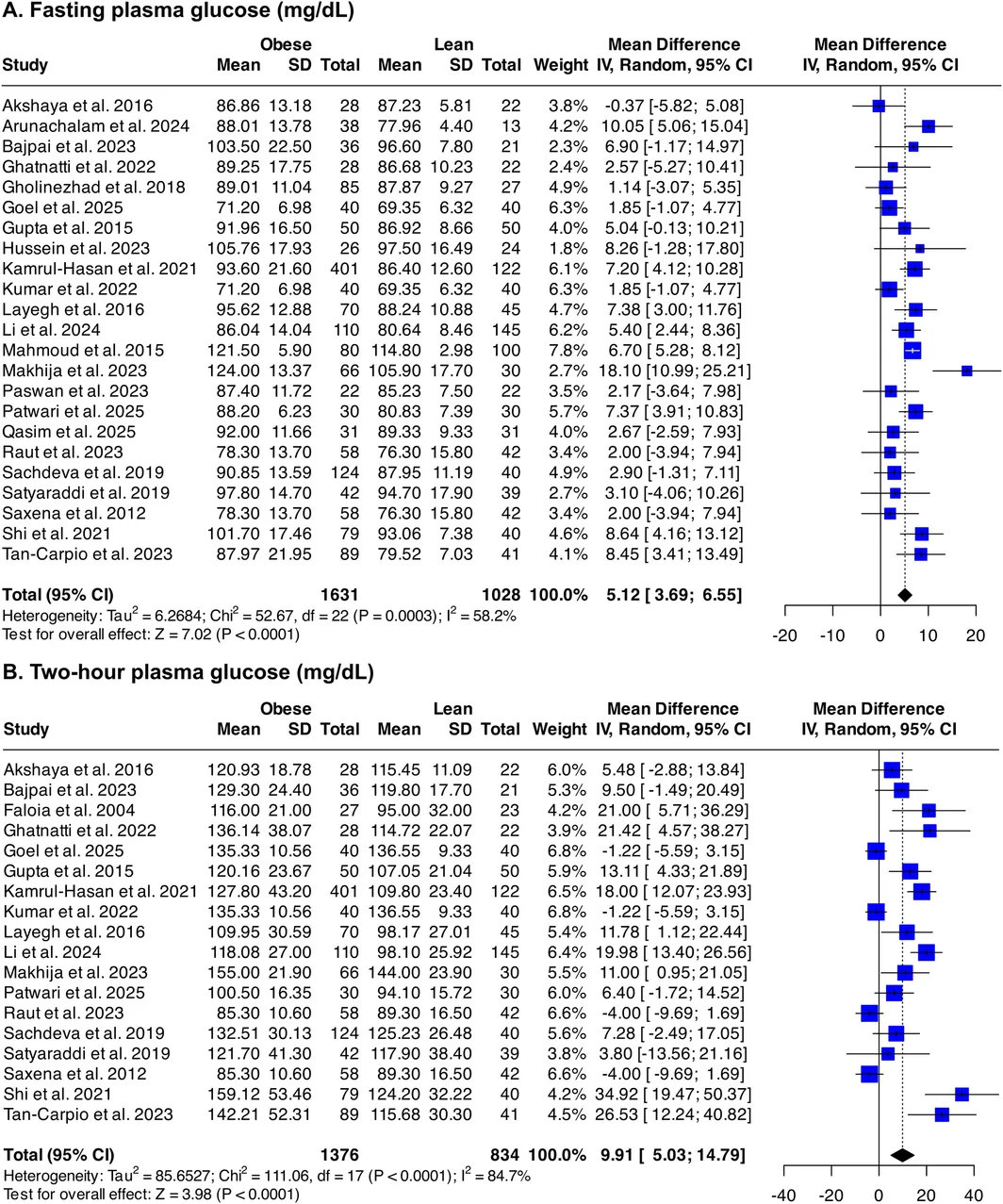
Forest plots of glycemic outcomes comparing obese versus lean women with polycystic ovary syndrome (PCOS). (A) Fasting plasma glucose (FPG), expressed as mg/dL. (B) Two‐hour plasma glucose (2 h PG) after an oral glucose tolerance test or breakfast, expressed as mg/dL. Pooled mean differences (MDs) were calculated using random‐effects inverse‐variance meta‐analysis and are presented with 95% confidence intervals (CIs). The vertical line of no effect represents MD = 0. Estimates to the right of the line indicate higher glucose values in the obese‐PCOS group, whereas estimates to the left indicate higher glucose values in the lean‐PCOS group.

### 3.13 Lipid Profile and Dyslipidemia

The obese PCOS group had higher total cholesterol (20 studies, *n* = 2427; MD 14.30 mg/dL [9.88; 18.72], *p* < 0.0001, *I* ^2^ = 88.4%; Figure 5A), triglycerides (22 studies, *n* = 2623; MD 27.73 mg/dL [19.12; 36.35], *p* < 0.0001, *I* ^2^ = 86.8%; Figure 5B), and low‐density lipoprotein cholesterol (LDL‐C) (17 studies, *n* = 2260; MD 12.02 mg/dL [9.14; 14.90], *p* < 0.0001, *I* ^2^ = 52.4%; Figure 5C) than the lean PCOS group. High‐density lipoprotein cholesterol (HDL‐C) was lower in the obese group (20 studies, *n* = 2506; MD −4.75 mg/dL [−7.54; −1.97], *p* = 0.0008, *I* ^2^ = 92.2%; Figure 5D). In contrast, the pooled frequency of categorically defined dyslipidemia, reported by only four studies (*n* = 853), was not significantly different between obese and lean PCOS (78.7% vs. 60.3%; RR 1.33 [0.95; 1.87], *p* = 0.0987, *I* ^2^ = 73.5%) (Table 1).

**FIGURE 5:**
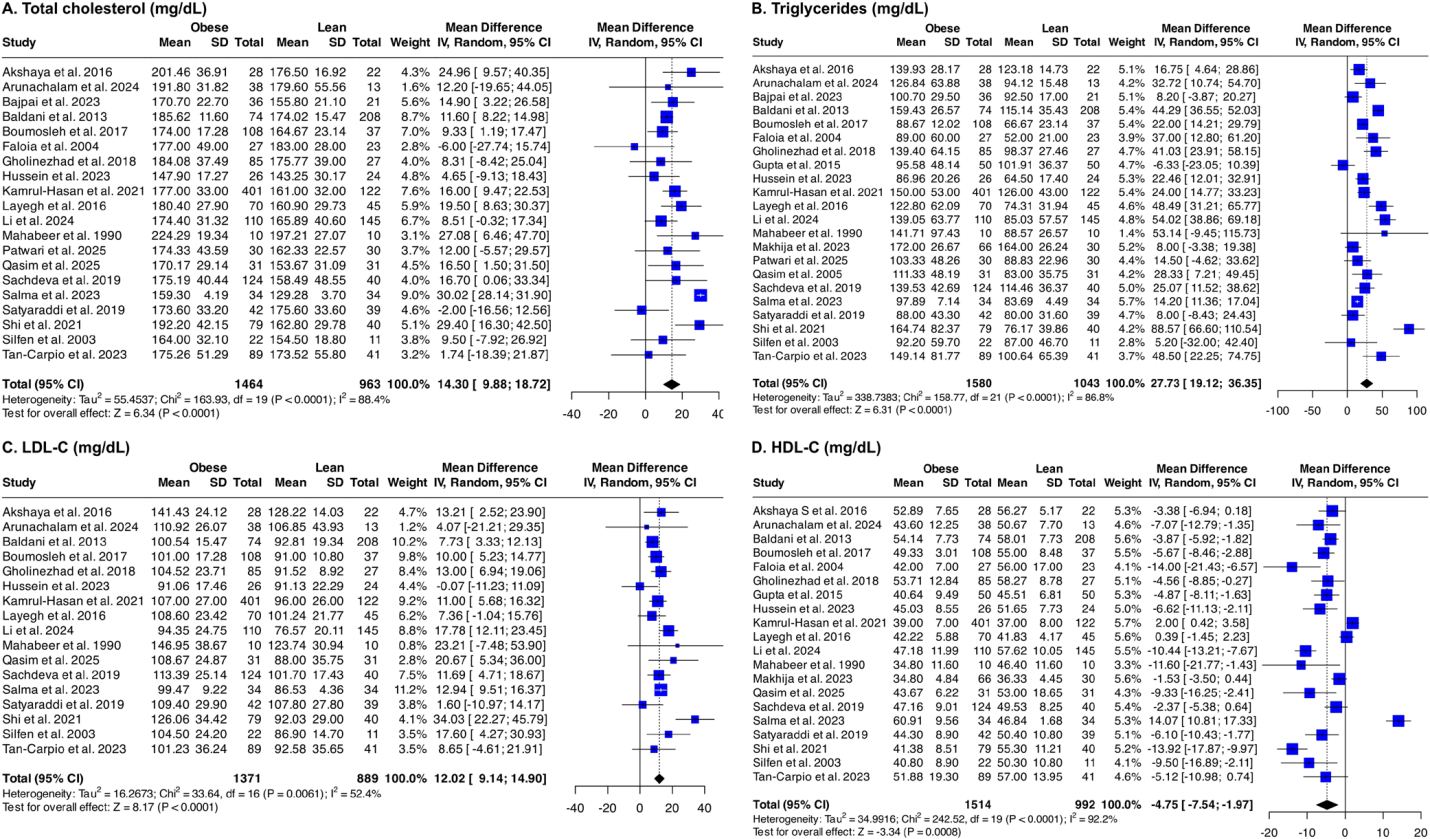
Forest plots of continuous lipid outcomes comparing obese versus lean women with polycystic ovary syndrome (PCOS). (A) Total cholesterol, expressed as mg/dL. (B) Triglycerides, expressed as mg/dL. (C) Low‐density lipoprotein cholesterol (LDL‐C), expressed as mg/dL. (D) High‐density lipoprotein cholesterol (HDL‐C), expressed as mg/dL. Pooled mean differences (MDs) were calculated using random‐effects inverse‐variance meta‐analysis and are presented with 95% confidence intervals (CIs). The vertical line of no effect represents MD = 0. Estimates to the right of the line indicate higher values in the obese‐PCOS group, whereas estimates to the left indicate higher values in the lean‐PCOS group.

### 3.14 Metabolic Syndrome

Metabolic syndrome (4 studies, *n* = 947) was more frequent in the obese than in the lean PCOS group (51.2% vs. 11.9%; RR 3.59 [2.50; 5.17], *p* < 0.0001, *I* ^2^ = 33.2%) (Table 1).

### 3.15 Publication Bias

Visual inspection of funnel plots and Egger's regression tests suggested small‐study effects for menstrual irregularity, TT, 2 h PG, total cholesterol, triglycerides, and HDL‐C (Figures S16–S18). These findings may reflect publication bias but should be interpreted cautiously, particularly given substantial between‐study heterogeneity.

## 4 Discussion

This comprehensive systematic review and meta‐analysis, synthesizing 42 studies and more than 5000 women with PCOS, suggests that excess adiposity substantially amplifies the clinical, metabolic, and hormonal severity of the syndrome and confirms that many adverse features are present even in lean phenotypes. Overall, obesity in PCOS is associated with more frequent menstrual disturbance, greater clinical and biochemical hyperandrogenism, more severe insulin resistance and dysglycemia, and a more atherogenic cardiometabolic profile, culminating in markedly higher rates of metabolic syndrome.

### 4.1 Summary of Main Findings

Across regions and BMI thresholds, women with PCOS and obesity exhibited significantly greater central adiposity than their lean counterparts, as indicated by higher waist circumference and waist–hip ratio. Menstrual irregularity and hyperandrogenic features were more common in the obese group, with higher risks of hirsutism, acne, and acanthosis nigricans, as well as increased circulating total testosterone. In contrast, mFG scores as continuous measures were broadly similar across phenotypes. Obesity was also linked to higher systolic and diastolic BP and more than double the risk of HTN, along with significantly increased HOMA‐IR, fasting and post‐load glucose levels, and notably higher risks of prediabetes and overt diabetes. Mean lipid concentrations were more adverse in obese PCOS, with higher total cholesterol, triglycerides, and LDL‐C and lower HDL‐C; however, the pooled prevalence of categorically defined dyslipidemia did not differ significantly between phenotypes. The prevalence of metabolic syndrome was more than threefold higher in obese than in lean PCOS. Importantly, lean women with PCOS still exhibited considerable burdens of menstrual dysfunction, hyperandrogenism, insulin resistance, dysglycemia, and dyslipidemia, underscoring that the absence of obesity does not equate to a benign phenotype.

### 4.2 Impact of Obesity on PCOS Severity

The findings indicate that adiposity is associated with a more adverse reproductive, endocrine, and cardiometabolic expression of PCOS, although it is not required for the syndrome to occur. This interpretation should remain non‐causal because the included studies were predominantly cross‐sectional and incompletely accounted for confounding by lifestyle, socioeconomic factors, medication exposure, and treatment washout. Nevertheless, the consistent direction of associations across multiple outcomes supports considering body size and fat distribution in phenotype assessment and risk stratification.

The reproductive and androgen‐related differences are biologically plausible. The higher prevalence of menstrual irregularity, hirsutism, acne, acanthosis nigricans, and biochemical hyperandrogenism in obese PCOS is consistent with hyperinsulinemia, altered adipokine signalling, reduced sex hormone‐binding globulin, increased ovarian androgen production, and disrupted hypothalamic–pituitary–ovarian function [4, 5, 6]. Acanthosis nigricans was particularly consistent, with an approximately twofold higher prevalence and no observed statistical heterogeneity, supporting its utility as a readily identifiable marker of metabolic risk. Conversely, similar mean modified Ferriman–Gallwey scores despite more frequent hirsutism may reflect variable diagnostic cutoffs, cultural and observer differences in hair scoring, and dichotomization of continuous scores.

The cardiometabolic differences may be clinically meaningful. Obese women with PCOS had substantially higher blood pressure and more than twice the rate of hypertension, consistent with data linking visceral adiposity to endothelial dysfunction and sympathetic activation [3, 4, 5]. HOMA‐IR and categorical insulin resistance were higher in obese PCOS and were accompanied by higher fasting and 2‐h plasma glucose concentrations and higher prevalences of prediabetes and diabetes. These associations indicate a more adverse glycemic profile in obese PCOS [3, 4, 5]. Mean lipid concentrations were also less favourable in obese PCOS, with higher total cholesterol, triglycerides, and LDL‐C and lower HDL‐C. In contrast, categorically defined dyslipidemia was not significantly more prevalent, probably because only four studies contributed to this outcome, definitions and thresholds varied, and heterogeneity was substantial.

Very high heterogeneity across HOMA‐IR, total testosterone, 2 h PG, and several lipid outcomes limits the precision and generalizability of pooled estimates. Variable BMI thresholds, PCOS diagnostic criteria, age composition (including adolescent cohorts), ethnicity, outcome definitions, laboratory methods, and treatment exposure may have contributed. Exploratory subgroup analyses by BMI cutoff, geographic region, and diagnostic criteria provide context but cannot resolve residual confounding or establish individual‐level effect modification. Funnel‐plot asymmetry and Egger's regression tests suggested small‐study effects for several outcomes, indicating that some pooled estimates may be inflated. These results should therefore be interpreted as directional associations rather than precise causal effect sizes.

### 4.3 Adverse Features in Lean PCOS

Lean PCOS should not be considered mild or metabolically benign. Despite lower average burdens across several outcomes than in obese PCOS, lean women still had substantial menstrual dysfunction, clinical hyperandrogenic symptoms, insulin resistance, dysglycemia, and metabolic syndrome. More than two‐thirds experienced menstrual irregularity, over half had hirsutism, and more than one‐third had acne. The observed prevalence of prediabetes and diabetes, despite normal or low BMI, indicates that anthropometry alone cannot reliably identify clinically meaningful metabolic risk.

Adverse features in lean PCOS may reflect intrinsic neuroendocrine dysregulation, increased GnRH pulse frequency and LH secretion, altered ovarian steroidogenesis, genetic susceptibility, ectopic or visceral adiposity not captured by BMI, and developmental influences such as prenatal androgen exposure [4, 6, 7]. These mechanisms are biologically plausible but cannot be tested or ranked using aggregate observational data. Thus, the findings support a heterogeneous PCOS spectrum in which generalized obesity may amplify risk, whereas clinically relevant metabolic and androgen‐related abnormalities may persist at normal BMI.

Lipid findings warrant caution. Categorically defined dyslipidemia was reported in approximately 60% of lean PCOS participants across four studies, but its pooled prevalence did not differ significantly from that of obese PCOS. This estimate should not be generalized because dyslipidemia definitions varied and heterogeneity was substantial. Differences in total testosterone were more apparent in Asian cohorts and in studies using the Rotterdam or modified Rotterdam criteria; these exploratory patterns underscore how population characteristics, diagnostic frameworks, and assay methods shape observed PCOS phenotypes.

### 4.4 Clinical Implications

The findings support phenotype‐informed PCOS care rather than a binary distinction between “lean” and “obese” disease. In obese PCOS, the more adverse average cardiometabolic profile and higher prevalence of several adverse outcomes support routine evaluation of blood pressure, glycemia, lipids, and other cardiometabolic risk factors. International guidance recommends lifestyle management for all women with PCOS, integrating weight‐management strategies, anti‐obesity pharmacotherapy where appropriate, and metabolic surgery in selected individuals with obesity, alongside reproductive and psychological care [60]. This meta‐analysis supports those principles but does not establish the effectiveness of interventions or treatment thresholds in either phenotype.

Normal BMI should not preclude metabolic assessment. In lean PCOS, evaluation of glycemic status, blood pressure, lipid profile, and other risk factors should be individualized based on clinical presentation, family history, ethnicity, and guideline recommendations. Counselling should prioritize sustainable healthy eating, physical activity, cardiorespiratory fitness, prevention of weight gain, and limiting central adiposity rather than weight loss per se. Management of menstrual dysfunction, hyperandrogenism, infertility, insulin resistance, or dysglycemia should follow guideline‐based indications rather than assumptions of low risk in lean PCOS.

Counselling should convey both phenotypic differences and their overlap. In women with obesity, weight reduction may improve reproductive and metabolic outcomes, although this cross‐sectional meta‐analysis cannot quantify the effect size. In lean PCOS, normal weight does not preclude metabolic vulnerability; periodic reassessment is appropriate with weight gain, worsening hyperandrogenic symptoms, abnormal glycemic findings, or a family history of diabetes.

### 4.5 Strengths and Limitations

This meta‐analysis has several notable strengths. First, to our knowledge, it is the largest quantitative comparison of lean and obese PCOS phenotypes, involving 42 studies across multiple continents and healthcare settings, including over 5000 women. Second, the review followed a prospectively registered protocol and adhered to Cochrane and PRISMA 2020 standards, used clear eligibility criteria, and implemented standard processes for study selection, data extraction, and quality assessment, using a current JBI tool for analytical cross‐sectional studies. Third, it examined a wide range of clinical, hormonal, and metabolic outcomes, providing a comprehensive view of how adiposity influences the PCOS phenotype rather than concentrating on individual biomarkers. Nonetheless, important limitations remain. All included studies were observational, mainly cross‐sectional, which limit causal inference and increase the risk of residual confounding by lifestyle, socioeconomic, or treatment‐related factors that were not consistently reported, measured, or adjusted for. Definitions of obesity and leanness varied, with BMI cut‐offs ranging from 23 to 30 kg/m^2^ and different use of Asian‐specific versus WHO thresholds. Several included cohorts focused on adolescents, while others enrolled mixed adolescent and adult populations. We did not conduct age‐restricted meta‐analyses, so including adolescents, who generally have shorter disease duration, may bias pooled metabolic estimates downward and contribute to heterogeneity. Future work should explicitly examine age‐stratified phenotypes. Additionally, PCOS was diagnosed using multiple criteria sets, which may contribute to clinical heterogeneity. Outcome definitions and laboratory methods for measuring testosterone, insulin, and lipids, as well as thresholds for insulin resistance, dyslipidemia, and metabolic syndrome, were not standardized. This limits the ability to explore dose–response relationships or phenotype‐specific thresholds. Most studies used heterogeneous immunoassays and rarely reported free testosterone, sex hormone‐binding globulin, or the free androgen index, so total testosterone is an imperfect surrogate that likely underestimates or misclassifies androgen excess across BMI strata. Therefore, we view obesity as a modifier rather than a direct cause of hyperandrogenism in PCOS. For outcomes with substantial between‐study heterogeneity (notably HOMA‐IR, TT, and several lipid fractions), the pooled estimates should be treated as directional estimates of association and approximate effect magnitude rather than as precise quantitative effects. Although subgroup analyses by BMI cutoff, geographic region, and diagnostic criteria partially contextualized this variability, we did not conduct formal meta‐regression, so marked heterogeneity persisted. Because subgroup analyses were study‐level, based on a limited number of studies for some outcomes, and not supported by meta‐regression, they should be interpreted as exploratory and cannot establish effect modification at the individual‐patient level. We did not perform outcome‐specific sensitivity analyses; therefore, we could not formally assess the robustness of the pooled estimates to excluding individual studies, lower‐quality studies, or other influential data points. Moreover, funnel‐plot asymmetry and Egger's regression tests suggested small‐study effects for menstrual irregularity, total testosterone, 2 h PG, total cholesterol, triglycerides, and HDL‐C; these findings may reflect publication bias and indicate that some pooled effects could be inflated. Most studies were conducted at specialist centers in South and West Asia and the Middle East, with limited data from other regions, which may limit generalizability across ethnicities and healthcare systems. The cross‐sectional evidence does not allow assessment of long‐term cardiovascular or diabetes outcomes by BMI phenotype, highlighting the need for large, well‐phenotyped prospective cohorts. Since this review aimed to compare obese and lean PCOS phenotypes, it did not incorporate a pooled healthy non‐PCOS control group. Therefore, the findings describe relative differences between PCOS phenotypes but cannot quantify their absolute excess reproductive or cardiometabolic burden relative to women without PCOS. Finally, because all eligible studies and the prespecified protocol used the historical term PCOS, we retained this terminology rather than the newly proposed term PMOS. This may limit alignment with evolving nomenclature but does not affect the eligibility criteria, pooled estimates, or interpretation of the findings.

Future research should prioritize prospective, age‐stratified cohorts using standardized definitions of BMI categories, metabolic outcomes, and PCOS phenotypes, and systematically assess visceral adiposity and metabolic dysfunction‐associated steatotic liver disease, given their potential relevance to the cardiometabolic phenotype of PCOS. Harmonized androgen assays, including reliable assessments of free testosterone and sex hormone‐binding globulin, and adequately designed studies of phenotype‐specific lifestyle, pharmacological, and reproductive interventions are needed. Preclinical studies may further investigate pathways linking adipose tissue dysfunction, insulin resistance, ovarian steroidogenesis, inflammation, and hepatic steatosis; however, the present observational meta‐analysis does not identify or validate specific therapeutic targets.

## 5 Conclusion

In summary, obesity in women with PCOS was associated with more frequent menstrual irregularity, clinical and biochemical hyperandrogenism, insulin resistance, dysglycemia, and a less favourable mean lipid profile. These associations were accompanied by a notably higher prevalence of metabolic syndrome. However, lean PCOS is also marked by notable reproductive and metabolic issues, indicating that a normal BMI does not confer protection and should not reduce the need for screening or long‐term follow‐up. These data support viewing PCOS as a spectrum in which adiposity significantly amplifies an underlying endocrine–metabolic vulnerability, and emphasize the importance of phenotype‐specific strategies that integrate reproductive goals with structured cardiometabolic risk reduction throughout life.

## Author Contributions

**Fatema Tuz Zahura Aalpona:** writing – original draft, data curation, methodology, software, investigation, formal analysis. **Apurba Sarker:** data curation, methodology, formal analysis, visualization, writing – review and editing. **Lutfun Nahar:** data curation, methodology, investigation, writing – review and editing, visualization. **Md Abdul Bari Robel:** methodology, software, investigation, validation, resources, visualization, writing – review and editing. **A. B. M. Kamrul‐Hasan:** conceptualization, methodology, writing – review and editing, writing – original draft, visualization, project administration, resources, supervision, validation.

## Funding

The authors did not receive any financial support for the research, authorship, or publication of this article.

## Conflicts of Interest

The authors declare no conflicts of interest.

## Supporting information


**Appendix S1:** Search strategy.
**Table S1:** Major characteristics of the included studies and study participants.
**Table S2:** Joanna Briggs Institute (JBI) critical appraisal checklist for analytical cross‐sectional studies.
**Figure S1:** Forest plot of the mean difference in baseline age (years) comparing obese versus lean women with polycystic ovary syndrome (PCOS). Pooled mean differences (MDs) were calculated using random‐effects inverse‐variance meta‐analysis and are presented with 95% confidence intervals (CIs). The vertical line of no effect represents MD = 0. Estimates to the right of the line indicate higher values in the obese PCOS group, whereas estimates to the left indicate higher values in the lean PCOS group.
**Figure S2:** Forest plot of the mean difference in the baseline body mass index (kg/m^2^) comparing obese versus lean women with polycystic ovary syndrome (PCOS). Pooled mean differences (MDs) were calculated using random‐effects inverse‐variance meta‐analysis and are presented with 95% confidence intervals (CIs). The vertical line of no effect represents MD = 0. Estimates to the right of the line indicate higher values in the obese PCOS group, whereas estimates to the left indicate higher values in the lean PCOS group.
**Figure S3:** Forest plot of the mean difference in baseline waist circumference (cm) comparing obese versus lean women with polycystic ovary syndrome (PCOS). Pooled mean differences (MDs) were calculated using random‐effects inverse‐variance meta‐analysis and are presented with 95% confidence intervals (CIs). The vertical line of no effect represents MD = 0. Estimates to the right of the line indicate higher values in the obese PCOS group, whereas estimates to the left indicate higher values in the lean PCOS group.
**Figure S4:** Forest plot of the mean difference in baseline waist‐to‐hip ratio comparing obese versus lean women with polycystic ovary syndrome (PCOS). Pooled mean differences (MDs) were calculated using random‐effects inverse‐variance meta‐analysis and are presented with 95% confidence intervals (CIs). The vertical line of no effect represents MD = 0. Estimates to the right of the line indicate higher values in the obese PCOS group, whereas estimates to the left indicate higher values in the lean PCOS group.
**Figure S5:** Forest plot of the mean difference in the modified Ferriman–Gallwey score (points) comparing obese versus lean women with polycystic ovary syndrome (PCOS). Pooled mean differences (MDs) were calculated using random‐effects inverse‐variance meta‐analysis and are presented with 95% confidence intervals (CIs). The vertical line of no effect represents MD = 0. Estimates to the right of the line indicate higher values in the obese PCOS group, whereas estimates to the left indicate higher values in the lean PCOS group. Subgroups were defined by body mass index (BMI) cut‐off, and the *p* value for subgroup differences tests whether pooled effects differed between subgroups.
**Figure S6:** Forest plot of the mean difference in the modified Ferriman–Gallwey score (points) comparing obese versus lean women with polycystic ovary syndrome (PCOS). Pooled mean differences (MDs) were calculated using random‐effects inverse‐variance meta‐analysis and are presented with 95% confidence intervals (CIs). The vertical line of no effect represents MD = 0. Estimates to the right of the line indicate higher values in the obese PCOS group, whereas estimates to the left indicate higher values in the lean PCOS group. Subgroups were defined by the geographic region/continent of the included studies (Asian versus Non‐Asian), and the *p* value for subgroup differences tests whether pooled effects differed between subgroups.
**Figure S7:** Forest plot of the mean difference in total testosterone levels (ng/dL) comparing obese versus lean women with polycystic ovary syndrome (PCOS). Pooled mean differences (MDs) were calculated using random‐effects inverse‐variance meta‐analysis and are presented with 95% confidence intervals (CIs). The vertical line of no effect represents MD = 0. Estimates to the right of the line indicate higher values in the obese PCOS group, whereas estimates to the left indicate higher values in the lean PCOS group. Subgroups were defined by body mass index (BMI) cut‐off, and the *p* value for subgroup differences tests whether pooled effects differed between subgroups.
**Figure S8:** Forest plot of the mean difference in total testosterone levels (ng/dL) comparing obese versus lean women with polycystic ovary syndrome (PCOS). Pooled mean differences (MDs) were calculated using random‐effects inverse‐variance meta‐analysis and are presented with 95% confidence intervals (CIs). The vertical line of no effect represents MD = 0. Estimates to the right of the line indicate higher values in the obese PCOS group, whereas estimates to the left indicate higher values in the lean PCOS group. Subgroups were defined by the geographic region/continent of the included studies (Asian versus Non‐Asian), and the *p* value for subgroup differences tests whether pooled effects differed between subgroups.
**Figure S9:** Forest plot of the mean difference in total testosterone levels (ng/dL) comparing obese versus lean women with polycystic ovary syndrome (PCOS). Pooled mean differences (MDs) were calculated using random‐effects inverse‐variance meta‐analysis and are presented with 95% confidence intervals (CIs). The vertical line of no effect represents MD = 0. Estimates to the right of the line indicate higher values in the obese PCOS group, whereas estimates to the left indicate higher values in the lean PCOS group. Subgroups were defined by the PCOS diagnostic criteria (Rotterdam/Modified Rotterdam versus Non‐Rotterdam) used in the included studies, and the *p* value for subgroup differences tests whether pooled effects differed between subgroups.
**Figure S10:** Forest plot of the mean difference in HOMA‐IR (units) comparing obese versus lean women with polycystic ovary syndrome (PCOS). Pooled mean differences (MDs) were calculated using random‐effects inverse‐variance meta‐analysis and are presented with 95% confidence intervals (CIs). The vertical line of no effect represents MD = 0. Estimates to the right of the line indicate higher values in the obese PCOS group, whereas estimates to the left indicate higher values in the lean PCOS group. Subgroups were defined by body mass index (BMI) cut‐off, and the *p* value for subgroup differences tests whether pooled effects differed between subgroups.
**Figure S11:** Forest plot of the mean difference in HOMA‐IR (units) comparing obese versus lean women with polycystic ovary syndrome (PCOS). Pooled mean differences (MDs) were calculated using random‐effects inverse‐variance meta‐analysis and are presented with 95% confidence intervals (CIs). The vertical line of no effect represents MD = 0. Estimates to the right of the line indicate higher values in the obese PCOS group, whereas estimates to the left indicate higher values in the lean PCOS group. Subgroups were defined by the geographic region/continent of the included studies (Asian versus Non‐Asian), and the *p* value for subgroup differences tests whether pooled effects differed between subgroups.
**Figure S12:** Forest plot of the mean difference in HOMA‐IR (units) comparing obese versus lean women with polycystic ovary syndrome (PCOS). Pooled mean differences (MDs) were calculated using random‐effects inverse‐variance meta‐analysis and are presented with 95% confidence intervals (CIs). The vertical line of no effect represents MD = 0. Estimates to the right of the line indicate higher values in the obese PCOS group, whereas estimates to the left indicate higher values in the lean PCOS group. Subgroups were defined by the PCOS diagnostic criteria (Rotterdam/Modified Rotterdam versus Non‐Rotterdam) used in the included studies, and the *p* value for subgroup differences tests whether pooled effects differed between subgroups.
**Figure S13:** Forest plot of the mean difference in fasting plasma glucose levels (mg/dL) comparing obese versus lean women with polycystic ovary syndrome (PCOS). Pooled mean differences (MDs) were calculated using random‐effects inverse‐variance meta‐analysis and are presented with 95% confidence intervals (CIs). The vertical line of no effect represents MD = 0. Estimates to the right of the line indicate higher values in the obese PCOS group, whereas estimates to the left indicate higher values in the lean PCOS group. Subgroups were defined by body mass index (BMI) cut‐off, and the *p* value for subgroup differences tests whether pooled effects differed between subgroups.
**Figure S14:** Forest plot of the mean difference in fasting plasma glucose levels (mg/dL) comparing obese versus lean women with polycystic ovary syndrome (PCOS). Pooled mean differences (MDs) were calculated using random‐effects inverse‐variance meta‐analysis and are presented with 95% confidence intervals (CIs). The vertical line of no effect represents MD = 0. Estimates to the right of the line indicate higher values in the obese PCOS group, whereas estimates to the left indicate higher values in the lean PCOS group. Subgroups were defined by the PCOS diagnostic criteria (Rotterdam/Modified Rotterdam versus Non‐Rotterdam) used in the included studies, and the *p* value for subgroup differences tests whether pooled effects differed between subgroups.
**Figure S15:** Forest plot of the risk ratio for diabetes mellitus prevalence comparing obese versus lean women with polycystic ovary syndrome (PCOS). Pooled risk ratios (RRs) were calculated using random‐effects inverse‐variance meta‐analysis and are presented with 95% confidence intervals (CIs). The vertical line of no effect represents RR = 1. Estimates to the right of the line indicate a higher risk or prevalence in the obese PCOS group, whereas estimates to the left indicate a higher risk or prevalence in the lean PCOS group. Subgroups were defined by the geographic region/continent of the included studies (Asian versus Non‐Asian), and the *p* value for subgroup differences tests whether pooled effects differed between subgroups.
**Figure S16:** Funnel plots assessing small‐study effects in studies comparing obese versus lean women with polycystic ovary syndrome (PCOS). (A) Menstrual irregularity, risk ratio (RR); (B) hirsutism, RR; (C) acne, RR; (D) acanthosis nigricans, RR; and (E) total testosterone, mean difference (MD; ng/dL). Each point represents an individual study. The horizontal axis shows the study effect estimate and the vertical axis shows its standard error. The vertical line represents the pooled effect estimate; the diagonal lines represent the approximate 95% pseudo‐confidence limits. Funnel‐plot asymmetry was assessed using Egger's regression test. Asymmetry may indicate small‐study effects, including possible publication bias, and should be interpreted cautiously in the presence of between‐study heterogeneity.
**Figure S17:** Funnel plots assessing small‐study effects in studies comparing obese versus lean women with polycystic ovary syndrome (PCOS). (A) Systolic blood pressure (BP), mean difference (MD; mmHg); (B) diastolic BP, MD (mmHg); (C) fasting plasma glucose (FPG), MD (mg/dL); (D) 2‐h plasma glucose (2 h PG), MD (mg/dL); and (E) prediabetes, risk ratio (RR). Each point represents an individual study. The horizontal axis shows the study effect estimate and the vertical axis shows its standard error. The vertical line represents the pooled effect estimate; the diagonal lines represent the approximate 95% pseudo‐confidence limits. Funnel‐plot asymmetry was assessed using Egger's regression test. Asymmetry may indicate small‐study effects, including possible publication bias, and should be interpreted cautiously in the presence of between‐study heterogeneity.
**Figure S18:** Funnel plots assessing small‐study effects in studies comparing obese versus lean women with polycystic ovary syndrome (PCOS). (A) Homeostasis model assessment of insulin resistance (HOMA‐IR), mean difference (MD; units); (B) total cholesterol, MD (mg/dL); (C) triglycerides, MD (mg/dL); (D) low‐density lipoprotein cholesterol (LDL‐C), MD (mg/dL); and (E) high‐density lipoprotein cholesterol (HDL‐C), MD (mg/dL). Each point represents an individual study. The horizontal axis shows the study effect estimate and the vertical axis shows its standard error. The vertical line represents the pooled effect estimate; the diagonal lines represent the approximate 95% pseudo‐confidence limits. Funnel‐plot asymmetry was assessed using Egger's regression test. Asymmetry may indicate small‐study effects, including possible publication bias, and should be interpreted cautiously in the presence of between‐study heterogeneity.

## Data Availability

All datasets created or examined in this research are included in this published article and its Supporting Information.
