# Supplementary material for: Comparison of Clinical, Metabolic, and Hormonal Profiles in Obese Versus Lean Women With Polycystic Ovary Syndrome: A Systematic Review and Meta‐Analysis
Source: Endocrinol Diabetes Metab. 2026 Oct 8;9(6):e70350. doi: 10.1002/edm2.70350 (PMC13650264; doi:10.1002/edm2.70350)
Supplement: Supplementary file 1 [file EDM2-9-e70350-s001.docx]

**Appendix S1. Search strategy**

**PubMed:**

(("Polycystic Ovary Syndrome"[Mesh] OR "Polycystic Ovarian Syndrome"[tiab] OR PCOS[tiab])

AND

("Obesity"[Mesh] OR obese*[tiab] OR overweight[tiab] OR "Body Mass Index"[Mesh] OR BMI[tiab])

AND

(lean*[tiab] OR "normal weight"[tiab] OR non-obese[tiab] OR nonobese[tiab] OR "normal BMI"[tiab])

AND

(clinical*[tiab] OR symptom*[tiab] OR hirsutism[tiab] OR acne[tiab] OR metabolic*[tiab] OR insulin*[tiab] OR glucose[tiab] OR lipid*[tiab] OR "insulin resistance"[tiab] OR hormonal[tiab] OR androgen*[tiab] OR testosterone[tiab] OR LH[tiab] OR FSH[tiab]))

**Scopus:**

TITLE-ABS-KEY ( ("Polycystic Ovary Syndrome" OR "Polycystic Ovarian Syndrome" OR PCOS)

AND ( "Obesity" OR obese* OR overweight OR "Body Mass Index" OR BMI )

AND ( lean* OR "normal weight" OR "non-obese" OR nonobese OR "normal BMI" )

AND ( clinical* OR symptom* OR hirsutism OR acne OR metabolic* OR insulin* OR glucose OR lipid* OR "insulin resistance" OR hormonal OR androgen* OR testosterone OR LH OR FSH ) )

**Web of Science:**

TS=( ("Polycystic Ovary Syndrome" OR "Polycystic Ovarian Syndrome" OR PCOS)

AND ( "Obesity" OR obese* OR overweight OR "Body Mass Index" OR BMI )

AND ( lean* OR "normal weight" OR non-obese OR nonobese OR "normal BMI" )

AND ( clinical* OR symptom* OR hirsutism OR acne OR metabolic* OR insulin* OR glucose OR lipid* OR "insulin resistance" OR hormonal OR androgen* OR testosterone OR LH OR FSH ) )

**Supplementary Table S1.** Major characteristics of the included studies and study participants

| **Study ID [Reference no]** | **Study type** | **Country** | **Major inclusion criteria** | **BMI cutoff (kg/m^2^) or Weight percentile for obese PCOS** | **N** | **Age (years),**  **mean±SD** | **BMI (kg/m^2^),**  **mean±SD** |
| --- | --- | --- | --- | --- | --- | --- | --- |
| Akshaya et al. 2016 [17],  Akshaya et al. 2016 [18] | Observational cross-sectional | India | Women of age range 15-38 years diagnosed to have PCOS according to ESHRE/ASRM criteria | 23 | Total 50 | NA | 25.9±4.5 |
|  |  |  |  |  | Lean 22 | NA | 21.9±1.2 |
|  |  |  |  |  | Obese 28 | NA | 29.5±2.4 |
| Arunachalam et al. 2024 [19] | Retrospective cross-sectional | India | Women aged 18-35 years, diagnosed with PCOS according to modified Rotterdam criteria of ESHRE and ASRM | NA | Total 51 | 24.9±4.3 | 29.1±6.5 |
|  |  |  |  |  | Lean 13 | NA | NA |
|  |  |  |  |  | Obese 38 | NA | NA |
| Bajpai et al. 2023 [20] | Prospective observational cross-sectional | India | Rotterdam criteria in patients aged 18-45 years and National Institute of Health (NIH) strict criteria for adolescent age group (15-17 years) | 23 | Total 57 | NA | NA |
|  |  |  |  |  | Lean 21 | NA | NA |
|  |  |  |  |  | Obese 36 | NA | NA |
| Baldani et al. 2013 [21] | Cross-sectional | Croatia | Women aged 18-38 years, diagnosed with PCOS based on the Rotterdam consensus criteria | 25 | Total 282 | 27±5.8 | 24.6±4.9 |
|  |  |  |  |  | Lean 208 | 27±5.8 | 22.4±1.8 |
|  |  |  |  |  | Obese 74 | 27.1±5.9 | 31±5.4 |
| Boumosleh et al. 2017 [22] | Cross-sectional | United States of America | Rotterdam consensus criteria | NA | Total 145 | NA | NA |
|  |  |  |  |  | Lean 37 | 39.7±2.3 | 24.1±1.9 |
|  |  |  |  |  | Obese 108 | 40.7±2.2 | 34.4±3.3 |
| Diana-Irina et al. 2025 [23] | Cross-sectional | Romania | Adolescent patients (11-20 years) with confirmed PCOS, fulfilling diagnostic criteria of Adolescent PCOS: Abnormal uterine bleeding and biochemical and/or clinical hyperandrogenism | ≥ 85 percentile | Total 68 | NA | NA |
|  |  |  |  |  | Lean 27 | NA | NA |
|  |  |  |  |  | Obese 41 | NA | NA |
| Faloia et al. 2004 [24] | Observational cross-sectional | Italy | Caucasian women with PCOS diagnosed according to National Institute of Health (NIH) criteria | 25 | Total 50 | 22±5 | NA |
|  |  |  |  |  | Lean 23 | 23±4.9 | 22±2 |
|  |  |  |  |  | Obese 27 | 21±4.8 | 32.6±5.3 |
| Ghatnatti et al. 2022 [25] | Cross-sectional | India | PCOS was diagnosed using the Rotterdam 2003 criteria | 23.5 | Total 50 | NA | NA |
|  |  |  |  |  | Lean 22 | 23.4±4.2 | 21.1±1 |
|  |  |  |  |  | Obese 28 | 24.5±4.2 | 26.2±2.8 |
| Gholinezhad et al. 2018 [26] | Comparative cross-sectional | Iran | Women aged 18-35 years with PCOS was diagnosed using the 2003 Rotterdam consensus criterion | 25 | Total 112 | NA | NA |
|  |  |  |  |  | Lean 27 | 22.4±3.5 | 22.4±2.3 |
|  |  |  |  |  | Obese 85 | 24.4±5 | 30.9±3.8 |
| Goel et al. 2025 [27] | Prospective comparative cross-sectional | India | All young women aged 18-22 years with PCOS fulfilling Rotterdam criteria | 25 | Total 80 | NA | NA |
|  |  |  |  |  | Lean 40 | 20.2±1.4 | 22.6±2.1 |
|  |  |  |  |  | Obese 40 | 20.4±1.5 | 28.4±2.5 |
| Gupta et al. 2022 [28] | Comparative cross-sectional | India | Women aged 18-35 years diagnosed using Rotterdam criteria 2003 | 23 | Total 80 | NA | NA |
|  |  |  |  |  | Lean 40 | NA | NA |
|  |  |  |  |  | Obese 40 | NA | NA |
| Gupta et al. 2015 [29] | Comparative cross-sectional | India | Women aged 18-35 years diagnosed using Rotterdam criteria 2003 | 23 | Total 100 | NA | NA |
|  |  |  |  |  | Lean 50 | NA | NA |
|  |  |  |  |  | Obese 50 | NA | NA |
| Hamadamen et al. 2017 [30] | Cross-sectional comparative | Iraq | Women with PCOS diagnosed using Rotterdam criteria | 25 | Total 230 | NA | NA |
|  |  |  |  |  | Lean 107 | 28.8±4.8 | 23.4±1.3 |
|  |  |  |  |  | Obese 123 | 29.5±5.9 | 32.9±4.3 |
| Hussein et al. 2023 [31] | Cross-sectional | Iraq | Any female patient who had previously been clinically diagnosed with PCOS and expressed willingness to participate | 30 | Total 50 | NA | 27.5±7.7 |
|  |  |  |  |  | Lean 24 | NA | NA |
|  |  |  |  |  | Obese 26 | NA | NA |
| Kala et al. 2013 [32] | Cross-sectional | India | Women aged between 12-42 years, who fulfilled the Rotterdam criteria | 23 | Total 100 | NA | NA |
|  |  |  |  |  | Lean 25 | NA | NA |
|  |  |  |  |  | Obese 75 | NA | NA |
| Kamrul-Hasan et al. 2021 [33] | Cross-sectional | Bangladesh | Adults and adolescents diagnosed with PCOS according to revised Rotterdam criteria and Endocrine Society criteria, respectively | 23 | Total 523 | 21.9±5.9 | 26.8±5.2 |
|  |  |  |  |  | Lean 122 | 20±3.9 | 20.3±1.9 |
|  |  |  |  |  | Obese 401 | 22.6±5.7 | 28.8±4.2 |
| Kayastha et al. 2023 [34] | Prospective cross-sectional | India | Women aged between 20-38 years and were diagnosed to have PCOS according to Rotterdam ESHRE/ASRM criteria | 23 | Total 100 | NA | NA |
|  |  |  |  |  | Lean 20 | NA | 22.4±0.9 |
|  |  |  |  |  | Obese 80 | NA | 30.9±2.9 |
| Kiddy et al. 1990 [35] | Cross-sectional | United Kingdom | Women who were found to have polycystic ovaries on ultrasound scanning. The patients had presented with either hirsutism or anovulation or both | 25 | Total 263 | NA | NA |
|  |  |  |  |  | Lean 172 | NA | 21±1.8 |
|  |  |  |  |  | Obese 91 | NA | 30.3±4.2 |
| Komarowska et al. 2013 [36] | Cross-sectional | Poland | Patients with diagnosed al ovarian syndrome according to Rotterdam criteria | 25 | Total 20 | 25±4.4 | 26.2±5.9 |
|  |  |  |  |  | Lean 10 | 25±4.3 | 21.5±1.2 |
|  |  |  |  |  | Obese 10 | 26±4.3 | 30±5.1 |
| Kumar et al. 2022 [37] | Prospective comparative cross-sectional | India | All young women 18-22 years of age with PCOS diagnosed according to Rotterdam criteria | 25 | Total 80 | NA | NA |
|  |  |  |  |  | Lean 40 | 20.2±1.4 | 22.6±2.1 |
|  |  |  |  |  | Obese 40 | 20.4±1.5 | 28.4±2.5 |
| Layegh et al. 2016 [38] | Cross-sectional | Iran | PCOS patients, age from 16-45 years were enrolled using the Rotterdam 2003 criteria for selection of the patients | 25 | Total 115 | 24.5±5.4 | NA |
|  |  |  |  |  | Lean 45 | 22.5±4.5 | NA |
|  |  |  |  |  | Obese 70 | 25.8±5.7 | NA |
| Li et al. 2024 [39] | Cross-sectional | China | Women with PCOS diagnosed according to Rotterdam criteria | 25 | Total 255 | NA | NA |
|  |  |  |  |  | Lean 145 | 24.2±4.6 | NA |
|  |  |  |  |  | Obese 110 | 25±4.8 | NA |
| Liou et al. 2009 [40] | Retrospective cross-sectional | Taiwan | Women who met the Rotterdam 2003 criteria were diagnosed with PCOS | 25 | Total 295 | 26.7±5.4 | 24.5±6.2 |
|  |  |  |  |  | Lean 180 | 26.2±5.2 | 20.3±2.1 |
|  |  |  |  |  | Obese 115 | 27.6±5.5 | 31.2±4.4 |
| Mahabeer et al. 1990 [41] | Comparative Observational cross-sectional | South Africa | Non-obese PCOD were selected on the basis of hirsutism, menstrual abnormalities, no acanthosis nigricans, serum LH:FSH >2 and BMI <24 kg/m^2^  Obese PCOD group had hirsutism, menstrual abnormalities, hyperandrogenism and a Serum LH:FSH > 2. | 24 | Total 20 | NA | NA |
|  |  |  |  |  | Lean 10 | 23.9±3.6 | 21±2.4 |
|  |  |  |  |  | Obese 10 | 23.7±6.7 | 29.7±3.1 |
| Mahmoud et al. 2015 [42] | Comparative cross-sectional | Saudi Arabia | All included women were previously diagnosed to have PCOS depending on their ultrasound findings by presence of eight or more subcapsular follicular cysts ≤ 10 mm and increased ovarian stroma | 25 | Total 180 | 41.7±6.8 | NA |
|  |  |  |  |  | Lean 100 | NA | NA |
|  |  |  |  |  | Obese 80 | NA | NA |
| Majumdar et al. 2009 [43] | Observational cross-sectional | India | Women satisfying the ESHRE and ASRM criteria for PCOS were included | 23 | Total 450 | NA | NA |
|  |  |  |  |  | Lean 150 | NA | NA |
|  |  |  |  |  | Obese 300 | NA | NA |
| Makhija et al. 2023 [44] | Observational cross-sectional | India | Consenting women between the ages of 15 and 45 with PCOS met the inclusion criteria, according to Rotterdam guidelines | 25 | Total 96 | 25.4±5.2 | 26.3±3.4 |
|  |  |  |  |  | Lean 30 | NA | NA |
|  |  |  |  |  | Obese 66 | NA | NA |
| Paswan et al. 2023 [45] | Case-control study | India | PCOS patients were recruited from the out-patient department, thyroid diseases and hyper-prolactinemia were excluded | 30 | Total 44 | NA | NA |
|  |  |  |  |  | Lean 22 | 25.1±5 | 21.3±1.5 |
|  |  |  |  |  | Obese 22 | 29.2±2.7 | 32±1.9 |
| Patwari et al. 2025 [46] | Cross-sectional | India | Women aged 18-40 who met the Rotterdam criteria 2003 for PCOS | 27 | Total 60 | NA | NA |
|  |  |  |  |  | Lean 30 | 26.2±3.7 | 20.8±1.9 |
|  |  |  |  |  | Obese 30 | 24.6±5.7 | 29.8±1.7 |
| Qasim et al. 2025 [47] | Cross-sectional | Iraq | Women diagnosed with polycystic ovary syndrome according to Rotterdam criteria | 30 | Total 62 | 24.9±6.1 | 27.7±5.2 |
|  |  |  |  |  | Lean 31 | NA | NA |
|  |  |  |  |  | Obese 31 | NA | NA |
| Raut et al. 2023 [48] | Cross-sectional | Kyrgyzstan | Women with age range 20-38 years presenting with infertility over a 3 year period and diagnosed to have PCOS according to ESHRE/ASRM/FOGSI criteria | 23 | Total 100 | NA | NA |
|  |  |  |  |  | Lean 42 | 28.4±4.2 | 22.3±4.1 |
|  |  |  |  |  | Obese 58 | 29±5.2 | 28.1±5.8 |
| Sachdeva et al. 2019 [49] | Prospective observational cross-sectional | India | Women aged <40 years with PCOS related infertility based on Rotterdam criteria | 23 | Total 164 | NA | NA |
|  |  |  |  |  | Lean 40 | 27.7±3.6 | NA |
|  |  |  |  |  | Obese 124 | 28±3.8 | NA |
| Salma et al. 2023 [50] | Retrospective cross-sectional | Saudi Arabia | All women who were diagnosed with PCOS by Rotterdam criteria | 25 | Total 68 | NA | NA |
|  |  |  |  |  | Lean 34 | 24.7±6.1 | 21.2±1.3 |
|  |  |  |  |  | Obese 34 | 26.7±8.3 | 30.5±5.7 |
| Satyaraddi et al. 2019 [51] | Cross-sectional | India | Women who were 18-35 years of age with phenotypic features of PCOS fulfilling Rotterdam criteria | 25 | Total 81 | NA | NA |
|  |  |  |  |  | Lean 39 | 25.4±3.8 | 22.2±2.4 |
|  |  |  |  |  | Obese 42 | 25.2±3.9 | 30.9±4.9 |
| Saxena et al. 2012 [52] | Cross-sectional | India | Women with age range 20-38 years presenting with infertility over a 3 year period and diagnosed to have PCOS according to ESHRE/ASRM criteria | 23 | Total 100 | NA | NA |
|  |  |  |  |  | Lean 42 | 28.4±4.2 | 22.3±4.1 |
|  |  |  |  |  | Obese 58 | 29±5.2 | 28.1±5.8 |
| Sangos et al. 2000 [53] | Comparative cross-sectional | Greece | Women with PCOS aged 18-27 years, no excessive exercise (>1 hour/day), no aggressive dieting(loss of >1 kg/week),no disease affecting gonadotropin or sex steroid hormone secretion | NA | Total 34 | NA | NA |
|  |  |  |  |  | Lean 17 | 21.7±3.4 | 22.6±1.7 |
|  |  |  |  |  | Obese 17 | 22.4±4.3 | 30.9±5.2 |
| Sharami et al. 2015 [54] | Cross-sectional | Iran | Married women with PCOS aging from 15 to 39 years, diagnosis based on Rotterdam criteria | 25 | Total 357 | NA | NA |
|  |  |  |  |  | Lean 157 | 25.6±4.7 | NA |
|  |  |  |  |  | Obese 200 | 26.8±5.3 | NA |
| Shi et al. 2021 [55] | Comparative cross-sectional | China | All patients with PCOS were at least 18 years of age, PCOS diagnosed according to Rotterdam PCOS diagnostic criteria | 28 | Total 119 | NA | NA |
|  |  |  |  |  | Lean 40 | NA | 22±3.6 |
|  |  |  |  |  | Obese 79 | NA | 30.5±4 |
| Siddiqui et al. 2009 [56] | Cross-sectional | Saudi Arabia | All patients between the age 29 and 43 years with PCOS fulfilling the revised 2003 Rotterdam criteria | 25 | Total 62 | 35.8±5 | 31±6.4 |
|  |  |  |  |  | Lean 22 | 34.7±4.6 | NA |
|  |  |  |  |  | Obese 40 | 36.5±5.2 | NA |
| Silfen et al. 2003 [57] | Cross-sectional | United States of America | For the PCOS subjects the criteria for entry into the study were the presence of hyperandrogenism and oligomenorrhea or amenorrhea | 25 | Total 33 | NA | NA |
|  |  |  |  |  | Lean 11 | 16.1±1.9 | 22.5±1.5 |
|  |  |  |  |  | Obese 22 | 15.5±1.4 | 35.9±6.2 |
| Tamimi et al. 2009 [58] | Cross-sectional | Saudi Arabia | The study patients were between the ages of 29 and 43 years and complaints of Infertility, menstrual dysfunction, and/or dermatologic problems suggestive of PCOS | 30 | Total 47 | 35.8±5 | 31.9±6.4 |
|  |  |  |  |  | Lean 7 | 33.1±4.4 | NA |
|  |  |  |  |  | Obese 40 | 36.5±5.1 | NA |
| Tan-Carpio et al. 2021 [59] | Cross-sectional | Philippine | Women of reproductive age(18-40 years) with PCOS diagnosed based on Rotterdam criteria | NA | Total 130 | 27±5.9 | 25.9±4.7 |
|  |  |  |  |  | Lean 41 | NA | NA |
|  |  |  |  |  | Obese 89 | NA | NA |

ASHRE, American Society of Human Reproductive Medicine; BMI, Body mass index; ESHRE, European Society of Human Reproduction and Embryology; FOGSI, Federation of Obstetrics and Gynecology Societies of India; NA, Not available; PCOD, Polycystic Ovarian disease; PCOS, Polycystic ovary syndrome; SD, Standard deviation.

**Supplementary Figure S1.** Forest plot of the mean difference in baseline age (years) comparing obese versus lean women with polycystic ovary syndrome (PCOS). Pooled mean differences (MDs) were calculated using random-effects inverse-variance meta-analysis and are presented with 95% confidence intervals (CIs). The vertical line of no effect represents MD = 0. Estimates to the right of the line indicate higher values in the obese PCOS group, whereas estimates to the left indicate higher values in the lean PCOS group.

**Supplementary Figure S2.** Forest plot of the mean difference in the baseline body mass index (kg/m^2^) comparing obese versus lean women with polycystic ovary syndrome (PCOS). Pooled mean differences (MDs) were calculated using random-effects inverse-variance meta-analysis and are presented with 95% confidence intervals (CIs). The vertical line of no effect represents MD = 0. Estimates to the right of the line indicate higher values in the obese PCOS group, whereas estimates to the left indicate higher values in the lean PCOS group.

**Supplementary Figure S3.** Forest plot of the mean difference in baseline waist circumference (cm) comparing obese versus lean women with polycystic ovary syndrome (PCOS). Pooled mean differences (MDs) were calculated using random-effects inverse-variance meta-analysis and are presented with 95% confidence intervals (CIs). The vertical line of no effect represents MD = 0. Estimates to the right of the line indicate higher values in the obese PCOS group, whereas estimates to the left indicate higher values in the lean PCOS group.

**Supplementary Figure S4.** Forest plot of the mean difference in baseline waist-to-hip ratio comparing obese versus lean women with polycystic ovary syndrome (PCOS). Pooled mean differences (MDs) were calculated using random-effects inverse-variance meta-analysis and are presented with 95% confidence intervals (CIs). The vertical line of no effect represents MD = 0. Estimates to the right of the line indicate higher values in the obese PCOS group, whereas estimates to the left indicate higher values in the lean PCOS group.

**Supplementary Table S2.** Joanna Briggs Institute (JBI) critical appraisal checklist for analytical cross-sectional studies

| Study | Were the criteria for inclusion in the sample clearly defined? | Were the study subjects & the setting described in detail? | Was the exposure measured in a valid & reliable way? | Were objective, standard criteria used for measurement of the condition? | Were confounding factors identified? | Were strategies to deal with confounding factors stated? | Were the outcomes measured in a valid & reliable way? | Was appropriate statistical analysis used? |
| --- | --- | --- | --- | --- | --- | --- | --- | --- |
| Akshaya et al. 2016 [17,18] | Yes | Yes | No | No | No | No | No | Yes |
| Arunachalam et al. 2024 [19] | Yes | Yes | Yes | No | No | No | No | Yes |
| Bajpai et al. 2023 [20] | Yes | Yes | Yes | Yes | No | No | Yes | Yes |
| Baldani et al. 2013 [21] | Yes | Yes | Yes | Yes | No | No | Yes | Yes |
| Boumosleh et al. 2017 [22] | Yes | No | Yes | Yes | No | No | Yes | Yes |
| Diana-Irina et al. 2025 [23] | Yes | Yes | No | No | No | No | Yes | Yes |
| Faloia et al. 2004 [24] | Yes | No | Yes | Yes | No | No | Yes | Yes |
| Ghatnatti et al. 2022 [25] | Yes | Yes | Yes | Yes | No | No | Yes | Yes |
| Gholinezhad et al. 2018 [26] | Yes | Yes | Yes | Yes | No | No | Yes | Yes |
| Goel et al. 2025 [27] | Yes | No | Yes | Yes | No | No | Yes | Yes |
| Gupta et al. 2015 [28] | Yes | Yes | Yes | Yes | No | No | Yes | Yes |
| Gupta et al. 2022 [29] | Yes | No | Yes | Yes | No | No | Yes | No |
| Hamadamen et al. 2017 [30] | Yes | Yes | Yes | Yes | No | No | No | No |
| Hussein et al. 2023 [31] | No | No | Yes | Yes | No | No | Yes | Yes |
| Kala et al. 2013 [32] | Yes | Yes | Yes | Yes | No | No | Yes | No |
| Kamrul-Hasan et al. 2021 [33] | Yes | Yes | Yes | Yes | No | No | Yes | Yes |
| Kayastha et al. 2023 [34] | Yes | Yes | No | No | No | No | No | Yes |
| Kiddy et al. 1990 [35] | Yes | Yes | Yes | Yes | No | No | Yes | No |
| Komarowska et al. 2013 [36] | Yes | No | No | No | No | No | Yes | Yes |
| Kumar et al. 2022 [37] | Yes | Yes | Yes | Yes | No | No | Yes | Yes |
| Layegh et al. 2016 [38] | Yes | Yes | No | Yes | No | No | Yes | Yes |
| Li et al. 2024 [39] | Yes | Yes | Yes | Yes | No | No | Yes | Yes |
| Liou et al. 2009 [40] | Yes | Yes | Yes | Yes | No | No | Yes | Yes |
| Mahabeer et al.1990 [41] | No | No | No | No | No | No | No | Yes |
| Mahmoud et al. 2015 [42] | No | Yes | No | No | No | No | No | Yes |
| Majumdar et al. 2009 [43] | Yes | No | Yes | Yes | No | No | Yes | Yes |
| Makhija et al. 2023 [44] | Yes | Yes | Yes | Yes | No | No | Yes | No |
| Paswan et al. 2023 [45] | No | Yes | Yes | Yes | No | No | Yes | Yes |
| Patwari et al. 2025 [46] | Yes | No | No | Yes | No | No | Yes | Yes |
| Qasim et al. 2025 [47] | Yes | No | Yes | Yes | No | No | Yes | Yes |
| Raut et al. 2023 [48] | Yes | No | No | Yes | No | No | Yes | No |
| Sachdeva et al. 2019 [49] | Yes | No | Yes | Yes | No | No | Yes | Yes |
| Salma et al. 2023 [50] | Yes | No | No | No | No | No | No | Yes |
| Satyaraddi et al. 2019 [51] | Yes | No | No | Yes | No | No | Yes | Yes |
| Saxena et al. 2012 [52] | Yes | No | Yes | Yes | No | No | Yes | Yes |
| Sangos et al. 2000 [53] | Yes | No | No | Yes | No | No | Yes | Yes |
| Sharami et al. 2015 [54] | Yes | Yes | No | Yes | No | No | Yes | Yes |
| Shi et al. 2021 [55] | Yes | Yes | No | No | No | No | No | Yes |
| Siddiqui et al. 2009 [56] | Yes | Yes | Yes | Yes | No | No | Yes | Yes |
| Silfen et al. 2003 [57] | Yes | Yes | No | Yes | No | No | Yes | Yes |
| Tamimi et al. 2009 [58] | Yes | Yes | No | No | No | No | Yes | Yes |
| Tan-Carpio et al. 2021 [59] | Yes | Yes | No | No | No | No | Yes | Yes |

**Supplementary Figure S5.** Forest plot of the mean difference in the modified Ferriman-Gallwey score (points) comparing obese versus lean women with polycystic ovary syndrome (PCOS). Pooled mean differences (MDs) were calculated using random-effects inverse-variance meta-analysis and are presented with 95% confidence intervals (CIs). The vertical line of no effect represents MD = 0. Estimates to the right of the line indicate higher values in the obese PCOS group, whereas estimates to the left indicate higher values in the lean PCOS group. Subgroups were defined by body mass index (BMI) cut-off, and the P value for subgroup differences tests whether pooled effects differed between subgroups.

**Supplementary Figure S6.** Forest plot of the mean difference in the modified Ferriman-Gallwey score (points) comparing obese versus lean women with polycystic ovary syndrome (PCOS). Pooled mean differences (MDs) were calculated using random-effects inverse-variance meta-analysis and are presented with 95% confidence intervals (CIs). The vertical line of no effect represents MD = 0. Estimates to the right of the line indicate higher values in the obese PCOS group, whereas estimates to the left indicate higher values in the lean PCOS group. Subgroups were defined by the geographic region/continent of the included studies (Asian versus Non-Asian), and the P value for subgroup differences tests whether pooled effects differed between subgroups.

**Supplementary Figure S7.** Forest plot of the mean difference in total testosterone levels (ng/dL) comparing obese versus lean women with polycystic ovary syndrome (PCOS). Pooled mean differences (MDs) were calculated using random-effects inverse-variance meta-analysis and are presented with 95% confidence intervals (CIs). The vertical line of no effect represents MD = 0. Estimates to the right of the line indicate higher values in the obese PCOS group, whereas estimates to the left indicate higher values in the lean PCOS group. Subgroups were defined by body mass index (BMI) cut-off, and the P value for subgroup differences tests whether pooled effects differed between subgroups.

**Supplementary Figure S8.** Forest plot of the mean difference in total testosterone levels (ng/dL) comparing obese versus lean women with polycystic ovary syndrome (PCOS). Pooled mean differences (MDs) were calculated using random-effects inverse-variance meta-analysis and are presented with 95% confidence intervals (CIs). The vertical line of no effect represents MD = 0. Estimates to the right of the line indicate higher values in the obese PCOS group, whereas estimates to the left indicate higher values in the lean PCOS group. Subgroups were defined by the geographic region/continent of the included studies (Asian versus Non-Asian), and the P value for subgroup differences tests whether pooled effects differed between subgroups.

**Supplementary Figure S9.** Forest plot of the mean difference in total testosterone levels (ng/dL) comparing obese versus lean women with polycystic ovary syndrome (PCOS). Pooled mean differences (MDs) were calculated using random-effects inverse-variance meta-analysis and are presented with 95% confidence intervals (CIs). The vertical line of no effect represents MD = 0. Estimates to the right of the line indicate higher values in the obese PCOS group, whereas estimates to the left indicate higher values in the lean PCOS group. Subgroups were defined by the PCOS diagnostic criteria (Rotterdam/Modified Rotterdam versus Non-Rotterdam) used in the included studies, and the P value for subgroup differences tests whether pooled effects differed between subgroups.

**Supplementary Figure S10.** Forest plot of the mean difference in HOMA-IR (units) comparing obese versus lean women with polycystic ovary syndrome (PCOS). Pooled mean differences (MDs) were calculated using random-effects inverse-variance meta-analysis and are presented with 95% confidence intervals (CIs). The vertical line of no effect represents MD = 0. Estimates to the right of the line indicate higher values in the obese PCOS group, whereas estimates to the left indicate higher values in the lean PCOS group. Subgroups were defined by body mass index (BMI) cut-off, and the P value for subgroup differences tests whether pooled effects differed between subgroups.

**Supplementary Figure S11.** Forest plot of the mean difference in HOMA-IR (units) comparing obese versus lean women with polycystic ovary syndrome (PCOS). Pooled mean differences (MDs) were calculated using random-effects inverse-variance meta-analysis and are presented with 95% confidence intervals (CIs). The vertical line of no effect represents MD = 0. Estimates to the right of the line indicate higher values in the obese PCOS group, whereas estimates to the left indicate higher values in the lean PCOS group. Subgroups were defined by the geographic region/continent of the included studies (Asian versus Non-Asian), and the P value for subgroup differences tests whether pooled effects differed between subgroups.

**Supplementary Figure S12.** Forest plot of the mean difference in HOMA-IR (units) comparing obese versus lean women with polycystic ovary syndrome (PCOS). Pooled mean differences (MDs) were calculated using random-effects inverse-variance meta-analysis and are presented with 95% confidence intervals (CIs). The vertical line of no effect represents MD = 0. Estimates to the right of the line indicate higher values in the obese PCOS group, whereas estimates to the left indicate higher values in the lean PCOS group. Subgroups were defined by the PCOS diagnostic criteria (Rotterdam/Modified Rotterdam versus Non-Rotterdam) used in the included studies, and the P value for subgroup differences tests whether pooled effects differed between subgroups.

**Supplementary Figure S13.** Forest plot of the mean difference in fasting plasma glucose levels (mg/dL) comparing obese versus lean women with polycystic ovary syndrome (PCOS). Pooled mean differences (MDs) were calculated using random-effects inverse-variance meta-analysis and are presented with 95% confidence intervals (CIs). The vertical line of no effect represents MD = 0. Estimates to the right of the line indicate higher values in the obese PCOS group, whereas estimates to the left indicate higher values in the lean PCOS group. Subgroups were defined by body mass index (BMI) cut-off, and the P value for subgroup differences tests whether pooled effects differed between subgroups.

**Supplementary Figure S14.** Forest plot of the mean difference in fasting plasma glucose levels (mg/dL) comparing obese versus lean women with polycystic ovary syndrome (PCOS). Pooled mean differences (MDs) were calculated using random-effects inverse-variance meta-analysis and are presented with 95% confidence intervals (CIs). The vertical line of no effect represents MD = 0. Estimates to the right of the line indicate higher values in the obese PCOS group, whereas estimates to the left indicate higher values in the lean PCOS group. Subgroups were defined by the PCOS diagnostic criteria (Rotterdam/Modified Rotterdam versus Non-Rotterdam) used in the included studies, and the P value for subgroup differences tests whether pooled effects differed between subgroups.

**Supplementary Figure S15.** Forest plot of the risk ratio for diabetes mellitus prevalence comparing obese versus lean women with polycystic ovary syndrome (PCOS). Pooled risk ratios (RRs) were calculated using random-effects inverse-variance meta-analysis and are presented with 95% confidence intervals (CIs). The vertical line of no effect represents RR = 1. Estimates to the right of the line indicate a higher risk or prevalence in the obese PCOS group, whereas estimates to the left indicate a higher risk or prevalence in the lean PCOS group. Subgroups were defined by the geographic region/continent of the included studies (Asian versus Non-Asian), and the P value for subgroup differences tests whether pooled effects differed between subgroups.

**Supplementary Figure S16.** Funnel plots assessing small-study effects in studies comparing obese versus lean women with polycystic ovary syndrome (PCOS). (A) Menstrual irregularity, risk ratio (RR); (B) hirsutism, RR; (C) acne, RR; (D) acanthosis nigricans, RR; and (E) total testosterone, mean difference (MD; ng/dL). Each point represents an individual study. The horizontal axis shows the study effect estimate and the vertical axis shows its standard error. The vertical line represents the pooled effect estimate; the diagonal lines represent the approximate 95% pseudo-confidence limits. Funnel-plot asymmetry was assessed using Egger’s regression test. Asymmetry may indicate small-study effects, including possible publication bias, and should be interpreted cautiously in the presence of between-study heterogeneity.

**Supplementary Figure S17.** Funnel plots assessing small-study effects in studies comparing obese versus lean women with polycystic ovary syndrome (PCOS). (A) Systolic blood pressure (BP), mean difference (MD; mmHg); (B) diastolic BP, MD (mmHg); (C) fasting plasma glucose (FPG), MD (mg/dL); (D) 2-hour plasma glucose (2h PG), MD (mg/dL); and (E) prediabetes, risk ratio (RR). Each point represents an individual study. The horizontal axis shows the study effect estimate and the vertical axis shows its standard error. The vertical line represents the pooled effect estimate; the diagonal lines represent the approximate 95% pseudo-confidence limits. Funnel-plot asymmetry was assessed using Egger’s regression test. Asymmetry may indicate small-study effects, including possible publication bias, and should be interpreted cautiously in the presence of between-study heterogeneity.

**Supplementary Figure S18.** Funnel plots assessing small-study effects in studies comparing obese versus lean women with polycystic ovary syndrome (PCOS). (A) Homeostasis model assessment of insulin resistance (HOMA-IR), mean difference (MD; units); (B) total cholesterol, MD (mg/dL); (C) triglycerides, MD (mg/dL); (D) low-density lipoprotein cholesterol (LDL-C), MD (mg/dL); and (E) high-density lipoprotein cholesterol (HDL-C), MD (mg/dL). Each point represents an individual study. The horizontal axis shows the study effect estimate and the vertical axis shows its standard error. The vertical line represents the pooled effect estimate; the diagonal lines represent the approximate 95% pseudo-confidence limits. Funnel-plot asymmetry was assessed using Egger’s regression test. Asymmetry may indicate small-study effects, including possible publication bias, and should be interpreted cautiously in the presence of between-study heterogeneity.
